# Recent Advances on Graphene Quantum Dots for Bioimaging Applications

**DOI:** 10.3389/fchem.2020.00424

**Published:** 2020-06-03

**Authors:** Muhammad Rizwan Younis, Gang He, Jing Lin, Peng Huang

**Affiliations:** Marshall Laboratory of Biomedical Engineering, International Cancer Center, Laboratory of Evolutionary Theranostics (LET), School of Biomedical Engineering, Shenzhen University Health Science Center, Shenzhen, China

**Keywords:** graphene quantum dots, synthesis method, bioimaging, fluorescence imaging, two-photon fluorescence imaging

## Abstract

Being a zero-dimensional (0D) nanomaterial of the carbon family, graphene quantum dots (GQDs) showed promising biomedical applications owing to their ultra-small size, non-toxicity, biocompatibility, excellent photo stability, tunable fluorescence, and water solubility, etc., thus capturing a considerable attention in biomedical field. This review summarizes the recent advances made in the research field of GQDs and place special emphasis on their bioimaging applications. We briefly introduce the synthesis strategies of GQDs, including top–down and bottom–up strategies. The bioimaging applications of GQDs are also discussed in detail, including optical [fluorescence (FL)], two-photon FL, magnetic resonance imaging (MRI), and dual-modal imaging. In the end, the challenges and future prospects to advance the clinical bioimaging applications of GQDs have also been addressed.

## Introduction

Undoubtedly, the discovery of green fluorescence protein (GFP) and the development of organic fluorophores have fundamentally frameshifted the landscape of biomedical research, but their limited photostability made long-term bioimaging a formidable challenge (Zheng et al., 2015). Although semiconductor quantum dots (QDs) have emerged as a potential alternative to the developed organic fluorophores because of their photostability and brightness (Michalet et al., 2005; Baker, 2010), the intrinsic toxicity, poor water solubility, and blinking characteristics restricted their widespread imaging applications (Zhao et al., 2018; Lu et al., 2019). Additionally, compared to biological molecules, the larger-size semiconductor QDs (usually >500 kDa) might also affect the function and dynamics of target molecules (Zheng et al., 2015). Hence, the inherent limitations of organic and inorganic QD-based fluorophores gave birth to the critical and constant efforts, exploring state-of-the-art fluorophores for bioimaging applications.

**Figure 1:**
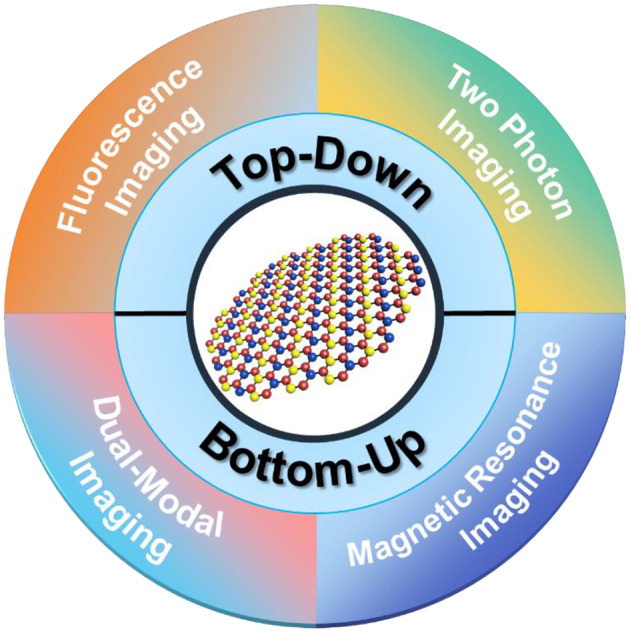
Graphene quantum dots (GQDs) for bioimaging applications. The synthesis strategies of GQDs, including top–down and bottom–up are summarized, followed by a comparative and balanced discussion on their bioimaging (fluorescence imaging, two-photon imaging, magnetic resonance imaging, and dual-modal imaging) applications.

Graphene quantum dots (GQDs), a latest zero-dimensional (0D) member of the carbon family, consist of single to few layers of graphene sheets with lateral dimensions of <10 nm (Li et al., 2013a; Benítez-Martínez and Valcárcel, 2015). Typically, GQDs not only possess the intriguing properties derived from two-dimensional (2D) graphene but also demonstrate extraordinary physicochemical characteristics of the QDs, including edge effects, non-zero band gap, and quantum confinement effects, by which they hold great potential in energy, electronic, and optical industry (Wang et al., 2016b; Chen et al., 2017a). In 2010, Pan et al. reported the successful synthesis of blue luminescent GQDs *via* hydrothermal route by cutting graphene sheets and discovering their fluorescent properties for the first time (Pan et al., 2010). Indeed, this work triggered innumerable experimental studies, and thus, a boom in GQD research has been witnessed to explore their potential bioimaging applications. Interestingly, owing to the excellent photostability, extended fluorescence, small size, biocompatibility, low cost, ease of preparation, non-toxicity, and water dispersibility, fluorescent GQDs surpass the conventional organic and semiconductor QD-based fluorophores, and emerged as a versatile and universal fluorophore, offering unprecedented opportunities in bioimaging for precise diagnosis (Shen et al., 2012; Li et al., 2017; Zhang and Ding, 2018). Though physicochemical properties, fluorescence mechanism, and applications of GQDs in energy, photocatalysis, optoelectronic devices (Haque et al., 2018; Tian et al., 2018; Yan et al., 2019), sensing (Fan et al., 2015; Zhou et al., 2016; Ozhukil Valappil et al., 2017), and cancer theranostics (Lin et al., 2016; Schroeder et al., 2016) have been well-reviewed, and the rapid advancements in bioimaging applications of GQDs strictly demand a periodic update. Therefore, in this mini review, we attempt to spotlight the latest developments in GQD research, focusing on their bioimaging applications. The different synthesis strategies of GQDs, including top–down and bottom–up are briefly summarized, followed by a comparative and balanced discussion on their bioimaging (fluorescence imaging, two-photon imaging, magnetic resonance imaging, and dual-modal imaging) applications (Figure 1). Last, future perspectives to overcome the existing bottlenecks are also highlighted. We envision that this review will offer a thorough understanding about the great promise of GQDs in bioimaging, which may assist in stimulating novel ideas, and hence, ultimately facilitate to push the GQD research to a climax.

## Synthesis Strategies of GQDs

Though a number of well-established fabrication methods of GQDs existed, the synthesis strategies are generally divided into two main categories, including “top–down” and “bottom–up.” In the top–down strategy, the bulk carbon materials such as graphene, carbon black, etc., are usually cleaved by chemical/electrochemical exfoliation, hydro/solvothermal treatment, and microwave/ultrasonication, resulting in nanoscale GQDs. Although the top–down strategy is highly suitable for mass production because of the abundant precursor materials and simple operation, the non-selective chemical cutting leads to poor control over the size and morphology of the ultimate product. Alternatively, the bottom–up strategy is based on the gradual growth of small precursor molecules (cyclic molecules, polymers) into nanosized GQDs by carbonization, pyrolysis, chemical vapor deposition, etc., offering high controllability and fewer defects. However, the poor solubility and aggregation of the resultant product is the main limitation, which needs careful consideration. In the following sub-sections, we will elaborate different synthesis methods underlying the umbrella of either top–down or bottom–up strategy.

### Top–Down Strategy

#### Chemical Exfoliation

**Figure 2:**
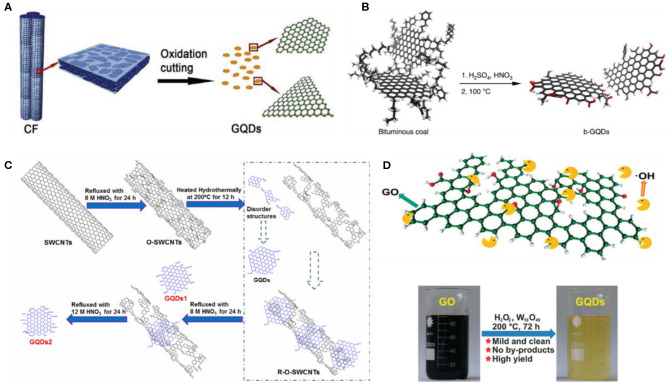
Diagrammatical representation of the acidic exfoliation of different carbon-based precursor such as **(A)** carbon fiber (CF); reproduced from Peng et al. (2012) with permission from the American Chemical Society. **(B)** Bituminous coal, reproduced from Ye et al. (2013) with permission from the Nature Publishing Group. **(C)** Single-walled CNTs (SWCNTs), reproduced from (Dong et al., 2013) with permission from Elsevier. **(D)** GO; reproduced from Zhu et al. (2015a) with permission from the Royal Society of Chemistry.

Chemical exfoliation method involves the exfoliation of precursor carbon materials such as graphene oxide (GO), carbon nanotubes (CNTs), carbon fibers, etc., by strong oxidizing agents and acids. It is a facile, straightforward, and cheap synthesis approach for the mass production of high-quality GQDs. Peng et al. prepared GQDs by exfoliating carbon fibers with a mixture of strong sulfuric acid (H_2_SO_4_) and nitric acid (HNO_3_) (Figure 2A). Owing to different stirring temperatures (80, 100, and 120°C), the resultant GQDs were in the size range of 1–11 nm with blue, green, and yellow emission, respectively, whereas the atomic force microscopy revealed the height of GQDs at around 0.4–2 nm, suggesting single to few graphene layers. It is notable to mention that the chemical cleavage of the sp^2^ domain of carbon fiber actually determines the successful formation of GQDs (Peng et al., 2012). Later, being an abundant and the cheapest material, Ye et al. utilized coal (anthracite, coke, and bituminous) as a precursor material to fabricate GQDs (Figure 2B). Under acidic cleavage of coal, hexagonal GQDs within a size range of 3–6 nm were obtained. Interestingly, they suggested that the structure of coal possesses crystalline carbon, which is highly suitable to undergo oxidative displacement, leading to the formation of GQDs (Ye et al., 2013). Subsequently, chemical exfoliation of graphite (Liu et al., 2013a) and asphaltene (Zhao et al., 2016a) was also reported to prepare GQDs with excitation-dependent photoluminescence. Though the combination of two strong acids (H_2_SO_4_ and HNO_3_) effectively exfoliated the precursor materials, the removal of excess sulfuric acid to purify the final product is a tedious process, which increases the overall synthesis cost.

Later, Dong et al. demonstrated that the concentrated HNO_3_ alone is sufficient to perform acidic cleavage of single-walled CNTs (SWCNTs), resulting in single to few layers of GQDs (Figure 2C) (Dong et al., 2013). Following this, an acid vapor cutting approach was reported by Xu et al., using an extremely low volume (2 ml) of HNO_3_ to cleave metal–organic framework-derived carbon, and the resultant GQDs were easily collected *via in situ* filtration. As the direct contact between the precursor and the oxidizing agent is strictly avoided, this method offers cheap and rapid synthesis of GQDs without any laborious purification process (Xu et al., 2015). In another study, graphene oxide (GO) was exfoliated by HNO_3_, following PEGylation and the reduction of exfoliated GO by hydrazine hydrate, yielding GQDs with an average diameter of 13.3 nm (Shen et al., 2011). Owing to the surface passivation, GQDs exhibit high fluorescence and up-conversion properties with a photoluminescence (PL) quantum yield (QY) of 7.4%.

Similarly, Kwon et al. reported the acidic cleavage of graphite using HNO_3_, followed by amidative cutting to fabricate GQDs, exhibiting colorful PL (Kwon et al., 2014). They suggested that the size of GQDs could easily be tuned by simply adjusting the amine concentration. Instead of HNO_3_, Maiti et al. employed perchloric acid to perform acidic exfoliation of GO to fabricate GQDs (Maiti et al., 2017). Notably, the prepared GQDs display strong excitation-independent PL with a QY of 14%, indicating the defect-free GQDs. On the other hand, spectroscopic analysis revealed that the conjugated peripheral functional groups with the planes of carbon backbone significantly contributed toward the excitation-independent PL property of the GQDs. Meanwhile, the longer PL life time (10 ns) as determined by the correlated single-photon counting (TCSPC) conferred the great potential of GQD for biological probing.

Besides acid-based oxidizing agents, powerful oxidants have also been reported for the chemical exfoliation/oxidation of carbon materials. For instance, Kundu's group chemically oxidized multiwalled CNTs (MWCNTs) using potassium permanganate (KMnO_4_) as an oxidant (Kundu et al., 2015a). The prepared GQDs were enriched with carboxyl (COOH) and hydroxyl (OH) functional groups, and possessed varying size ranges from 10 to 15 nm with an average hydrodynamic diameter of 12 nm. Instead of MWCNTs, Nair et al. oxidized GO by KMnO_4_ to prepare high-quality GQDs with a fluorescence QY of 23.8% (Nair et al., 2017). They demonstrated one pot acid-free synthesis of GQDs with a production yield of >74%. Although acid-based oxidizing agents and powerful oxidants showed high efficiency, the effective removal of these oxidizing agents to purify the final product is a tedious and challenging task. Keeping this in mind, Shin et al. utilized non-acid mild oxidant such as oxone to exfoliate different naturally present carbon precursors (MWCNTs, graphite, charcoal, and carbon fiber), and the prepared GQDs exhibits blue fluorescence (Shin et al., 2015). Subsequently, another mild oxidant hydrogen peroxide (H_2_O_2_) was employed by Zhu et al. (2015a) to oxidize GO with the assistance of tungsten oxide (W_18_O_49_) nanowires, leading to the fabrication of GQDs (Figure 2D). They suggested that the size of the GQDs could be tuned by adjusting the concentration of both H_2_O_2_ and W_18_O_49_. Notably, as the reactions products were only GQDs and water, the resultant GQDs could be directly applied for bioimaging without any prior purification. Being an acid-free strategy, mild oxidant-based exfoliation does not require extensive purification, offering simple, and environment friendly fabrication of GQDs.

#### Electrochemical Exfoliation

**Figure 3:**
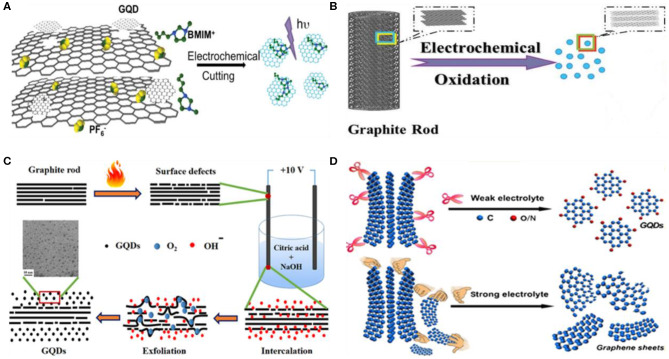
Illustration of GQD fabrication by electrochemical approach using 3D porous graphene **(A)**; reproduced from Ananthanarayanan et al. (2014), with permission from Wiley. **(B,C)** Graphite rods; reproduced from Su et al. (2015) and Ahirwar et al. (2017) with permission from Wiley and American Chemical Society. **(D)** Graphene paper; reproduced from Huang et al. (2018) with permission from the American Chemical Society.

Electrochemical cleavage of carbon-based precursors, such as graphene paper, coke, graphite, and CNTs, is a potential strategy, which has been broadly employed to prepare single-layer GQDs with uniform size and high production yield. Depending upon the electrolyte, the electrochemical exfoliation is generally divided into two sub-classes: water phase and organic phase electrochemical exfoliation (Zhou et al., 2016). By using organic electrolytes such as nitrogen-rich tetrabutylammonium perchlorate and acetonitrile, Li et al. prepared nitrogen-doped GQDs (N-GQDs) through an electrochemical cleavage of graphene film at a cyclic voltammetry (CV) scan rate of 0.5 V/s (Li et al., 2012c). The resultant N-GQDs were single to few layers thick as suggested by the topographical height (1–2.5 nm), around 2–5 nm in diameter, and possessed 4.3% atomic ratio of N/C. Owing to the heteroatom doping, N-GQDs displayed superior performance for electrocatalytic oxygen reduction reaction (OER) and high aqueous stability for long duration. In a subsequent study, 3D porous graphene was electrochemically oxidized by acetonitrile and 1-butyl-3-methylimidazolium hexafluorophosphate (BMIMPF_6_) (Figure 3A), leading to the fabrication of single-layer, highly crystalline, and uniform blue fluorescent GQDs with a hydrodynamic diameter of 3 nm (Ananthanarayanan et al., 2014). Because of the hexagonal crystalline structure and the presence of BMIM+ on the surface of GQDs, which contains electron-withdrawing N groups, these GQDs presented excitation-dependent PL with a QY of 10%.

As the safe disposal of organic electrolytes is a serious environmental concern, water phase electrooxidation of carbon-based precursors showed great promise to fabricate GQDs. For example, carbon fiber (CF) was electrochemically cleaved in borax (Na_2_B_4_O_7_·10H_2_O) electrolyte to prepare bright green fluorescent boron-doped GQDs (B-GQDs) (Fan et al., 2014). Another study demonstrated the formation of red fluorescent GQDs by electrochemically oxidizing graphite in potassium persulfate (K_2_S_2_O_8_, 0.01 M) (Tan et al., 2015). The persulfate (S_2_${\text{O}}_{8}^{2-}$) generated radicals (${\text{SO}}_{4}^{-}$) performed the sharp electrochemical scissoring of the graphene sheets, leading to the formation of GQDs with an average hydrodynamic diameter of 3 nm. Su et al. electrolyzed graphite rod in alkaline electrolyte (0.1 M NaOH), followed by reduction with hydrazine hydrate (Figure 3B). The resultant GQDs exhibited excitation-dependent fluorescence emission and were two to three layers thick as suggested by the atomic force microscopy (AFM) height profile (1–3 nm) and the dynamic light scattering (DLS) analysis (0.5–2.5 nm) (Su et al., 2015). Later, an accelerated electrochemical exfoliation of the graphite rod was presented by Li et al. using ultraviolet (UV) irradiation and H_2_O_2_ (Li et al., 2016c). They suggested that the electrons and the active radicals (HO· and H·) generated by UV irradiation and H_2_O_2_, effectively intercalated in the graphite working electrode and broke the C–C bond, thereby favoring the rapid generation of small-size GQDs with high yield.

Instead of single graphite rod and electrolysis, Bahadur's group prepared GQDs by thermally treating two graphite rods prior to electrochemical oxidation within an electrolyte containing both NaOH and citric acid (Ahirwar et al., 2017). The thermal treatment at 1,050°C induced several surface defects on graphite rods, which provided greater number of active sites to facilitate the electrochemical oxidation reaction (Figure 3C). The as-fabricated GQDs were around 2–3 nm in diameter and showed excitation-dependent PL property. Recently, weak electrolyte (ammonia solution, NH_4_OH)-mediated electrolysis was reported by Huang et al. for the first time (Huang et al., 2018). In their study, a graphene paper as a carbon precursor, completely oxidized within 120 min of electrochemical reaction, yielded highly crystalline GQDs with a product yield of 28%. The rapid electrochemical cutting is attributed to the low ionization capacity of the weak electrolyte, which effectively suppressed the intercalation. Second, due to the high concentration of the •OH radical, the weak electrolyte speeded up the electrochemical cutting and increased the overall reaction efficiency. During the electrochemical cleavage, the electrolyte solution rapidly turned from colorless to black due to the rapid formation of GQDs. In particular, they suggested that the strong electrolyte induced the rapid disintegration of graphite or graphene sheets, which leads to a low product yield, whereas the prolonged electrochemical cleavage and the suppression of intercalation by weak electrolytes result in an effective and steady fabrication of GQDs (Figure 3D). Impressively, this strategy could be further extended to other weak electrolytes, e.g., hydrogen fluoride (HF) and hydrogen sulfide (H_2_S), for the electrochemical cleavage of other carbon precursors, including CNTs, CF, graphite, and graphene.

#### Hydrothermal/Solvothermal Exfoliation

**Figure 4:**
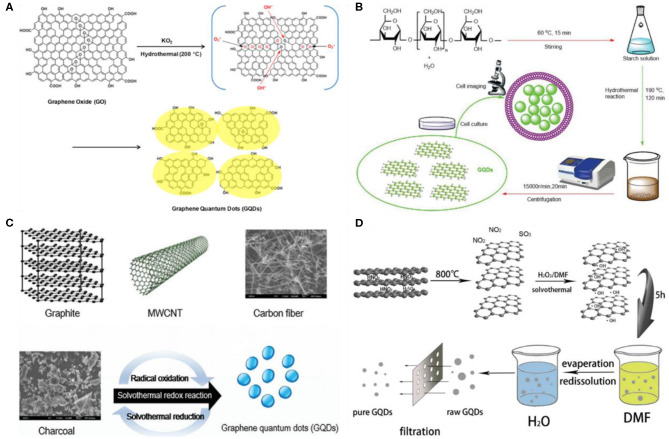
**(A)** Fabrication of GQDs by the hydrothermal exfoliation of GO with the assistance of potassium superoxide (KO_2_); reproduced from Zhao et al. (2017) with permission from Elsevier. **(B)** Hydrothermal cutting of starch to synthesize GQDs; reproduced from Chen et al. (2018) with permission from the Royal Society of Chemistry. **(C,D)** Acid-free solvothermal exfoliation of different carbon-based precursors to fabricate GQDs; reproduced from Shin et al. (2015) and Tian et al. (2016) with permission from the Royal Society of Chemistry and Elsevier.

Compared to other synthetic processes, hydro/solvothermal exfoliation is a simplified approach to prepare GQDs. For the first time, Pan et al. fabricated blue luminescent GQDs by the hydrothermal exfoliation of GO sheets (Pan et al., 2010). Prior to the thermal treatment, GO sheets were chemically oxidized, and thus, the resultant GO carbon lattice possessed several epoxy groups. Interestingly, these epoxy groups acted as a cleavage points and were completely broken during hydrothermal reaction to yield GQDs. Meanwhile, the simple and highly efficient approach (approximately 35 wt% conversion rate) to fabricate GQDs by hydrothermal treatment with the aid of potassium superoxide (KO_2_) was demonstrated by Zhao et al. (2017). The as-prepared water-soluble GQDs exhibited yellow emission with a photoluminescence QY of 8.9% (Figure 4A). Instead of chemical oxidation, GO sheets were fragmented by pulsed laser irradiation, followed by hydrothermal treatment to fabricate fluorescent GQDs with tunable emission (Qin et al., 2015a). Compared to chemical exfoliation, pulse laser-mediated ablation is a versatile and clean approach to prepare high-quality GQDs. To avoid the entire pre-processing step, Chen et al. reported on one pot hydrothermal exfoliation of starch to prepare GQDs (Chen et al., 2018). Owing to the absence of oxidizing agents, the extensive post-processing to purify the final product was avoided. Second, as the final reaction products were only GQDs and water (Figure 4B), the synthesis process is purely green and eco-friendly. Furthermore, the entire hydrothermal reaction was completed within 120 min, which suggested the rapid hydrothermal cutting of the precursor.

Alternatively, some researchers utilized oxidants or mild oxidants to accelerate the overall hydrothermal reaction. For instance, Halder et al. prepared GQDs by one pot hydrothermal exfoliation of GO sheets in the presence of H_2_O_2_ (Halder et al., 2018). Notably, H_2_O_2_ effectively scissors the GO sheets during thermal treatment and thus significantly accelerated the exfoliation reaction. The obtained GQDs were 5 nm in diameter, exhibited high photostability and non-cytotoxicity. Similarly, ascorbic acid-assisted hydrothermal cutting of graphite to synthesize GQDs was also demonstrated (Cirone et al., 2019). Recently, in contrast to the GQDs, Wang et al. prepared sulfur-doped GQDs (S-GQDs) by one pot hydrothermal exfoliation reaction (Wang et al., 2018a). Meanwhile, compared to the traditional carbon precursors for top–down GQD formation, they employed durian fruit as a precursor for the fabrication of S-GQDs. The fruit was first smashed and then hydrothermally treated at 150°C for 12 h in the presence of a platinum (Pt) sheet as a catalyst. The structure of the resultant S-GQDs were highly crystalline, while their size ranged from 2 to 6 nm with an average hydrodynamic diameter of 4 nm. The topographical height profile (0.5–1.0 nm) revealed single- to few-layer-thick S-GQDs. Owing to the S doping, S-GQDs displayed remarkable PL performance with a much higher PL quantum yield (79%) than most of the reported GQDs and extraordinary photo/chemical stability. Following the hydrothermal approach, N,S-co-doped GQDs (N,S-GQDs) were recently developed by Kulchat's group using citric acid as a source of carbon and cysteamine hydrochloride for nitrogen and sulfur, respectively (Boonta et al., 2020). The resultant N,S-co-doped GQD possessed an average hydrodynamic size of 3.0 nm with a size distribution of 1.1–5.4 nm. Though an excitation-dependent PL was observed, the co-doped GQDs were used for the fluorescence sensing of cobalt ions by metal–ligand interaction mechanism as cobalt ions interacted with carbon and the surface nitrogen and sulfur functional groups.

Apart from the hydrothermal fabrication, Zhu et al. reported the solvothermal approach to produce GQDs (Zhu et al., 2011). Green fluorescent GQDs with 11% fluorescence QY were formed by the solvothermal exfoliation of the GO sheets in dimethyl formamide (DMF) solvent for 5 h at 200°C. The single- or bilayer-thick GQDs as suggested by their height (1.2 nm) were about 5.3 nm in diameter. Following this, Wang's group also fabricated green fluorescent GQDs in DMF, but the obtained GQDs were a little bigger (4.92 nm) in size (Yu et al., 2017). Similar to oxidant-assisted hydrothermal cleavage, an oxone-assisted solvothermal exfoliation of different carbon precursors, including charcoal, MWCNTs, graphite, and CF (Figure 4C) was demonstrated by Shin et al. (2015). Later, Tian et al. presented mild oxidant (H_2_O_2_)-assisted solvothermal exfoliation of graphite (Figure 4D). Impressively, the non-acid oxidant-assisted solvothermal reaction do not require extensive dialysis for purification and thus exhibited facile, environment friendly, and low-cost fabrication of GQDs (Tian et al., 2016). During solvothermal reaction, the tunable PL of GQDs was studied by Qi et al. Following different reaction conditions, two different-sized GQDs (2.6 and 4.5 nm) with different surface chemistry were prepared (Qi et al., 2018). They suggested that the PL emission is largely influenced by the particle size and surface oxidation as both the larger-size particles and higher surface oxidation lead to bathochromic shift in the PL emission. Recently, Noor et al. performed solvothermal reaction in DMF and prepared white light-emitted pyrrolic N-doped GQDs (pN-GQDs) (Farain Md Noor et al., 2018). At a different reaction time (0.5–8 h), pN-GQDs, with a size range from 2.8 to 6.3 nm, were formed, while the concentration of pyrrolic N determined the luminescence properties of the resultant pN-GQDs as the brightness of the emitted white light increased with an increase in the concentration of pN-GQDs. Importantly, the concentration-dependent enhanced brightness feature is advantageous for the fabrication of white light-emitting diodes (LEDs) as well as for lighting applications.

#### Microwave/Ultrasound-Assisted Exfoliation

**Figure 5:**
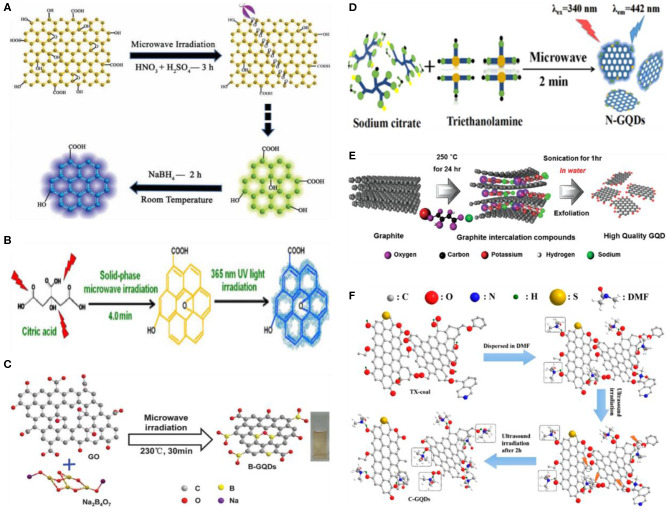
**(A)** Illustration of the preparation of GQDs by microwave-assisted chemical cleavage of GO sheets; reproduced from Li et al. (2012a) with permission from Wiley. **(B)** Microwave exfoliation of citric acid to fabricate GQDs; reproduced from Zhuang et al. (2016) with permission from Wiley. **(C)** Preparation of boron-doped GQDs (B-GQDs); reproduced from Hai et al. (2015) with permission from the Royal Society of Chemistry. **(D)** Preparation of nitrogen-doped GQDs (N-GQDs); reproduced from Ren et al. (2019) with permission from the American Chemical Society. **(E)** Ultrasonic preparation of GQDs using graphite; reproduced from Song et al. (2014) with permission from Wiley, and **(F)** coal as a precursor; reproduced from Zhang et al. (2019b) with permission from the American Chemical Society.

Being dependent on the conventional heating sources (oil bath, electric oven), the chemical/electrochemical and hydrothermal exfoliation methods usually suffer from long reaction time and, thus, are not suitable for the large-scale industrial production of GQDs. In contrast, microwave irradiation can remarkably shorten the reaction time by providing uniform heat, allowing the rapid formation of high-quality GQDs. Hence, the integration of microwave irradiation with other exfoliation approaches is an effective strategy to achieve a high yield of GQDs in less time. Li et al. for the first time reported on the preparation of GQDs by microwave-assisted chemical cleavage of GO sheets under acidic conditions (Li et al., 2012a). The acidic cleavage induced several epoxy groups on the GO sheets, which were easily ruptured under microwave treatment, leading to the formation of green fluorescent GQDs (Figure 5A). The height profile indicated single-layer GQDs with an average of 4.5 nm diameter. Interestingly, they further suggested that the blue fluorescent GQDs can be designed by the reduction of surface functional groups of green GQDs with sodium borohydride (NaBH_4_). By triggering the acidic cleavage and reduction simultaneously, microwave irradiation combined oxidation and reduction steps into a simple one step process toward GQD fabrication. Luo et al. reported the two-step fabrication of white fluorescent GQDs (Luo et al., 2016). First, graphite was transformed into yellowish green fluorescent GQDs under microwave-assisted acidic cleavage, followed by the microwave assisted hydrothermal (MAH) treatment of GQDs to yield white fluorescent GQDs. Apart from the chemical oxidation, a simple microwave exfoliation of citric acid (a common food additive) at 700 W for only 4 min was demonstrated to prepare GQDs as shown in Figure 5B (Zhuang et al., 2016). During the reaction, the solution turned from colorless to yellow depending upon the reaction time. Similarly, a time-dependent fluorescence emission was also noticed as a slight increase in the reaction time shifted the emission peak from 411 to 466 nm. Following this, the rapid preparation (3 min) of white fluorescent GQDs using trinitropyrene was also recently reported by Wu's group (Li et al., 2018c).

Later, different researchers employed the microwave-assisted method to prepare heteroatom-doped GQDs. Instead of microwave-assisted chemical cleavage, Sun et al. prepared fluorine-doped GQDs (F-GQDs) by the microwave-assisted hydrothermal (MAH) exfoliation of fluorinated GO (FGO) sheets (Sun et al., 2015). First, the FGO sheets were chemically cleaved in a mixture of strong oxidizing agents (HNO_3_/H_2_SO_4_), followed by microwave treatment at 650 W for 6 h. Similarly, the formation of F-GQDs using glucose as a precursor was demonstrated by Yang's group (Yousaf et al., 2017). The as-obtained F-GQDs were highly crystalline and smaller in size (2.38 ± 0.04 nm) than GQDs. Moreover, compared to GQDs, F-GQDs revealed green shift in PL, which is attributed to the fluorine doping. An acid-free doping of GQDs were reported by Hai et al. (2015). In their work, graphite and borax were used as a carbon and boron source, respectively, for the one-step microwave irradiation-mediated synthesis of boron-doped GQDs (B-GQDs) (Figure 5C). The as-prepared B-GQDs exhibited excitation-independent PL with a 21.1% QY.

Recently, Ren et al. prepared N-doped GQDs with a hydrodynamic size of 5.6 nm and a QY of 8% using both triethanolamine and sodium citrate as a precursor, respectively (Figure 5D) (Ren et al., 2019). In contrast to single-atom doping, Kundu et al. presented a simple strategy to develop multiatom (S, F, and N)-doped GQDs (Kundu et al., 2015b). In their study, MWCNTs as a carbon precursor were first dispersed in an ionic liquid (IL) *via* ultrasonication, followed by microwave treatment for 15 min at 1,100 W. After purification *via* dialysis and filtration, the multiatom-doped GQDs of around 2-nm size were obtained with high yield (78%). The rapid and efficient formation of heteroatom-doped GQDs was ascribed to the utilization of microwave-assisted strategy and an IL, which contained heteroatoms.

In addition to microwave-assisted exfoliation, ultrasound-assisted exfoliation have also been employed for the facile and mild fabrication of GQDs. In brief, ultrasound produces alternating high/low pressure waves in a liquid, which lead to the constant formation and abrupt collapse of small vacuum bubbles. Finally, these cavitations generated high-speed liquid jets, deagglomeration, and strong hydrodynamic shear forces (Li et al., 2011). Taking an advantage of these features, ultrasound is a favorable strategy to fracture the layered structures of carbon-based precursors into GQDs. In 2012; Zhuo et al., for the first time, prepared GQDs from the facile and direct ultrasonic exfoliation of graphene (Zhuo et al., 2012). Since then, a range of carbon materials such as cheap graphite, MWCNTs, CF, and GO have been explored for the ultrasonic preparation of GQDs in either organic solvent or aqueous solution (Zhou et al., 2016). Using graphite as a precursor, Song et al. reported the ultrasonic synthesis of GQDs (Song et al., 2014). In a typical experiment, graphite intercalation compounds (GICs) were first prepared by mixing potassium sodium tartrate and graphite in a mass ratio of 10:1, followed by hydrothermal treatment for 24 h at 250°C (Figure 5E). The aqueous dispersion of the as-synthesized GICs was further sonicated for an hour to produce GQDs. The resultant GQDs were about 1–5 nm in diameter and 0.5- to 1.5-nm thick as indicated by the height profile. Subsequently, Hassan et al. presented the direct ultrasonic exfoliation of few-layer graphene sheets (FLGs) and activated graphene to prepare GQDs (Hassan et al., 2014). The FLGs were transformed into activated graphene by treating with potassium hydroxide at a high temperature, followed by ultrasonication for 2 h to yield 16.3 wt% activated GQDs (aGQDs), while the yield of the GQDs produced by the direct sonication of FLGs was 3.4%. Following this, Zhang et al. recently fabricated blue luminescent GQDs from the one pot ultrasonic exfoliation of coal (anthracite) in DMF (Figure 5F). The as-obtained GQDs were highly stable, around 3.2 nm in diameter, and showed two fluorescence emission modes due to the surface defects and sp^2^ domain of carbon (Zhang et al., 2019b). Interestingly, ultrasonication approach also facilitated the fabrication of heteroatom-doped GQDs. For example, Zhao's group employed the chlorinated CF as a precursor for the ultrasonic formation of chlorine-doped GQDs (Cl-GQDs) in N-methylpyrrolidone (Zhao et al., 2015). In their work, hydrochloric acid (HCl) induced the chlorination of CF as well as cut the CF into small pieces. The resultant Cl-doped CF was then sonicated in N-methylpyrrolidone for 10 h to produce Cl-GQDs. As the heteroatom doping alters the physicochemical properties of the GQDs, the Cl-GQDs exhibited substantially improved photovoltaic performance.

### Bottom–Up Strategy

#### Carbonization/Pyrolysis

**Figure 6:**
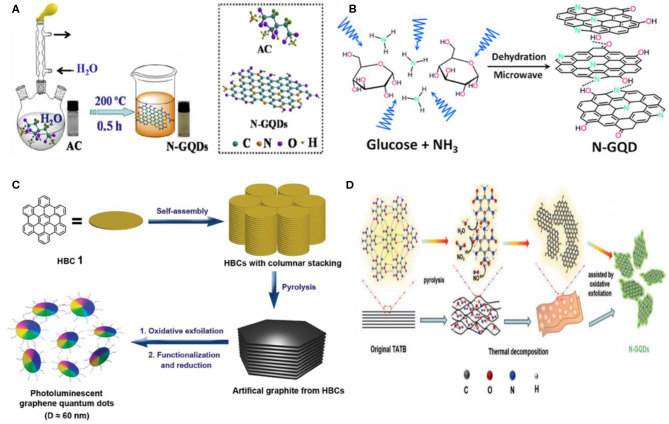
**(A)** Illustration of the carbonization process for GQD formation using ammonium citrate as a precursor; reproduced from Yin et al. (2016) with permission from Elsevier. **(B)** Pyrolysis of glucose; reproduced from Tang et al. (2014) with permission from the American Chemical Society. **(C)** Pyrolysis of hexa-peri-hexabenzocoronene (HBC) for GQDs; reproduced from Liu et al. (2011) with permission from the American Chemical Society. **(D)** Fabrication of N-GQDs using 1,3,5-triamino-2,4,6-trinitrobenzene (TATB) precursor; reproduced from Li et al. (2016b) with permission from Wiley.

The carbonization of small molecules/organic-based precursors is a simple and straightforward approach to fabricate GQDs, and thus have been widely explored in recent years. Specifically, the small organic-based precursor molecules are heated at a temperature higher than their melting point, which triggered the nucleation, condensation, and the subsequent fabrication of GQDs. A number of precursors, including organic salts, ethanolamine, acetylacetone, amino acids, co-factors (ascorbic acid), humic acid, coffee grounds, carbohydrates (sucrose or glucose), citric acid, etc., have been used for the bottom–up preparation of GQDs (Zhou et al., 2016). It is noteworthy to mention that the preparation of different types of GQDs, e.g., GQDs, heteroatom-doped GQDs, and heteroatom co-doped GQDs largely depends on the chosen precursor. For instance, HCl and fructose were chosen as a source of Cl and carbon to prepare Cl-GQDs (Li et al., 2013b). Under hydrothermal treatment, the O and H groups present within fructose were dehydrated, while the carbon formed the nucleus of GQDs. In the meantime, HCl catalyzed the reaction and offered a Cl dopant. Recently, Wang et al. synthesized highly fluorescent nitrogen-doped GQDs (N-GQDs) *via* direct carbonization using ammonium citrate as a precursor for both carbon and nitrogen, respectively (Yin et al., 2016). The homogenous aqueous solution of ammonium citrate was carbonized at high temperature (200°C), which led to the production of GQDs (Figure 6A). Following the similar concept, organic precursors were also carbonized to fabricate heteroatom-co-doped GQDs. In 2015, Qu's group fabricated nitrogen, sulfur-co-doped GQDs (N,S-GQDs) using thiourea and citric acid, respectively, as a precursor (Qu et al., 2015). Thiourea provides S and N atoms, while citric acid was used as a source of carbon. In their experiment, both the precursors were first efficiently dissolved in DMF, followed by thermal treatment at 180°C in a heating mantle. The purified GQDs were 1-nm thick, 4.5 in diameter, and exhibited excitation-dependent emission owing to the co-doping of N and S. Following the carbonization approach, the development of other heteroatom-doped GQDs such as P-GQDs, Si-GQDs, etc., is highly expected in the future.

For the pyrolysis-mediated fabrication of GQDs, the precursor molecules are generally divided into two categories: aromatic (hexa-peri-hexabenzocoronene, HBC) and non-aromatic (citric acid, glucose, etc.). Though the aromatic molecules possessed a pi system, the non-aromatic precursor molecules need intra- and intermolecular dehydrogenation (Ozhukil Valappil et al., 2017). Recently, Lee et al. fabricated single crystalline GQDs using D-glucose as a precursor (Lee et al., 2019). The as-prepared GQDs exhibited blue fluorescence, possessed hexagonal crystalline structure, and were 5 nm in lateral dimension. In contrast, using glucose as a precursor, N-GQDs were synthesized by Tang et al. in the presence of ammonia (NH_3_) (Tang et al., 2014). They suggested that NH_3_ not only doped N *in situ* but also performed the inter/intra-molecular dehydrogenation of glucose (Figure 6B). Owing to the resemblance of graphene fragments with the HBC and other polyaromatic hydrocarbons (PAH), the production of GQDs have also been demonstrated by the controlled pyrolysis of PAH-based precursor molecules as shown in Figure 6C (Liu et al., 2011). Besides PAH, a nitrogenous aromatic precursor such as melamine has been used by Xie's group to synthesize N-GQDs under high temperature (800–1,200°C) and pressure (4.0 GPa) (Zhu et al., 2015b). The resultant N-GQDs showed tunable band gap, possessed graphitic nature, and N-doping in the carbon lattice without any surface functional groups, which induced a negative effect on the electronic cloud (π electrons) of GQDs. Similarly, the formation of N-GQDs using citric acid and melamine as a carbon and N source, respectively, was also reported (Zhou et al., 2017). In another study, green fluorescent N-GQDs were formed using 1,3,5-triamino-2,4,6-trinitrobenzene (TATB) as an aromatic precursor (Figure 6D). It has been noticed that the intramolecular carbonization of TATB during pyrolysis plays a dominant role in the fabrication of N-GQDs (Li et al., 2016b). In contrast to high temperature/pressure carbonization, Habiba et al. transformed the benzene into GQDs by pulsed laser irradiation (Habiba et al., 2015). They suggested that under pulsed laser irradiation, the aromatic molecules generated free radicals, which first reorganized into carbon nanospheres and then combined together to yield GQDs. Meanwhile, the longer laser treatment yields bigger-size GQDs. Recently, a facile approach to design highly pure GQDs in the absence of any toxic chemical was presented by Lee et al. (2020). Briefly, they annealed SiC followed by etching with low vacuum H_2_ gas, resulting in highly crystalline and pure GQDs with fewer defects and the lack of surface functional groups of oxygen. Importantly, the regulation of vacuum pressure altered the morphology of the SiC surface, while the annealing temperature determined the size of the resultant GQDs as the decrease in annealing temperature from 1,500 to 1,400°C increased the size of GQDs from 2.58 to 5.20 nm.

#### Stepwise Organic Synthesis/Cage Opening

**Figure 7:**
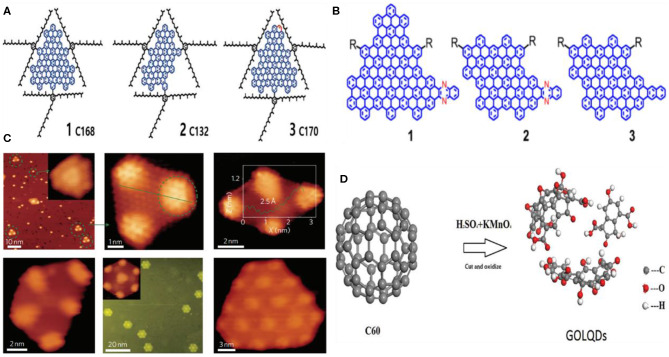
**(A)** GQDs containing different (170, 132, and 168) conjugated carbon atoms; reproduced from Yan et al. (2010) with permission from the American Chemical Society. **(B)** The representation of three different colloidal GQDs prepared by solution chemistry; reproduced from Li et al. (2012b) with permission from the American Chemical Society. **(C)** STM images represented the different shapes of GQDs prepared by cage opening approach; reproduced from Lu et al. (2011) with permission from Nature Publishing Group. **(D)** Illustration of the process of graphene-oxide-like QDs (GOLQDs) formation by the oxidization of C_60_; reproduced from Chen et al. (2015) with permission from Wiley.

Stepwise organic synthesis-mediated GQD fabrication is an effective solution chemistry method, which offers uniform and well-defined GQDs. Despite the significant advancements in the preparation of GQDs *via* stepwise organic synthesis, the poor aqueous solubility of the produced GQDs and the possibility of large molecular size because of the side reactions are the major bottlenecks, which need to be addressed (Ozhukil Valappil et al., 2017). Further, the low throughput and the aggregation of GQDs in solution due to π-π interactions also demanded careful considerations for industrial production. Mostly, the interaction of aliphatic side chains with the aromatic molecules brings the graphene sheets closer to each other, thus triggering the GQD aggregation (Haque et al., 2018). Therefore, the surface modification of the edges is an ideal way to avoid the aggregation by increasing the distance between graphene sheets, ultimately leading to improve the solubility of the GQDs. Following the solution chemistry method, Yan et al. used 3-iodo 4-bromo aniline as a precursor to fabricate three different-sized GQDs containing different (170, 132, and 168) conjugated carbon atoms (Figure 7A). They showed that the oxidative condensation of the precursor molecules during the stepwise process lead to fused the graphene moieties, resulting in the synthesis of GQDs, while, the modification of the edges by the covalent attachment of the 2′,4,6′-trialkyl phenyl groups allowed the fabrication of water-soluble GQDs (Yan et al., 2010). Notably, the covalent attachment of the phenyl groups at the edges significantly reduced the inter-layer distance of graphene as well as minimized the direct face to face contact between graphene sheets, allowing improved aqueous solubility and the fabrication of stable GQDs. Subsequently, the same group demonstrated the formation of heteroatom-doped GQDs (N-GQDs) by following the solution chemistry method (Li et al., 2012b). In their work, the N-GQDs were synthesized by a two-step process. First, a nitrogen-containing intermediate was prepared using small substituted benzene derivatives as a precursor such as phenylboronic acid and 2-bromo-5-iodo-30-(phenylethynyl)-1,10-biphenyl, followed by the Suzuki coupling of the intermediate molecule to form a GQD precursor. In the second step, the GQDs precursor was treated with an excess amount of iron (III) chloride under an inert environment (argon, Ar) in a mixture of nitromethane/dichloromethane to yield N-GQDs (Figure 7B). The as-obtained N-doped GQDs showed excellent electrocatalytic ORR activity.

Interestingly, the possibility of graphene wrapping into quasi 0-D fullerene GQDs provided a new concept to develop well-ordered GQDs from the fullerene *via* cage opening. For instance, Lu et al. used fullerene as a precursor and ruthenium (Ru) as a catalyst to rupture the C_60_ cage, leading to the formation of GQDs (Lu et al., 2011). As a reaction process, the thermal annealing of C_60_ at 500–550 K triggered the adatom-vacancy mechanisms, by which the molecules experience the dissociation and thermal hopping at the terrace as suggested by the scanning tunneling microscopy, while the C_60_ molecules fully decomposed at 650 K and transformed into uniform GQDs. They suggested that the annealing temperature determined the final shape of the GQDs as thermal annealing at 725 K for 2 min resulted in trapezoid-shaped, parallelogram-shaped, and triangular-shaped GQDs (Figure 7C), whereas the hexagonal GQDs of around 5–10 nm in diameter were obtained after thermal annealing at 825 K for an additional 2 min. Similarly, Chen et al. oxidized C_60_ molecules following a modified Hummers method and achieved ≈25 wt% yield of hexagonal graphene-oxide-like QDs (GOLQDs) (Figure 7D). The as-prepared GOLQDs were 0.6–2.2 nm in diameter, possessed an average thickness of 1.2 nm, and showed improved water solubility (Chen et al., 2015).

#### Chemical Vapor Deposition

**Figure 8:**
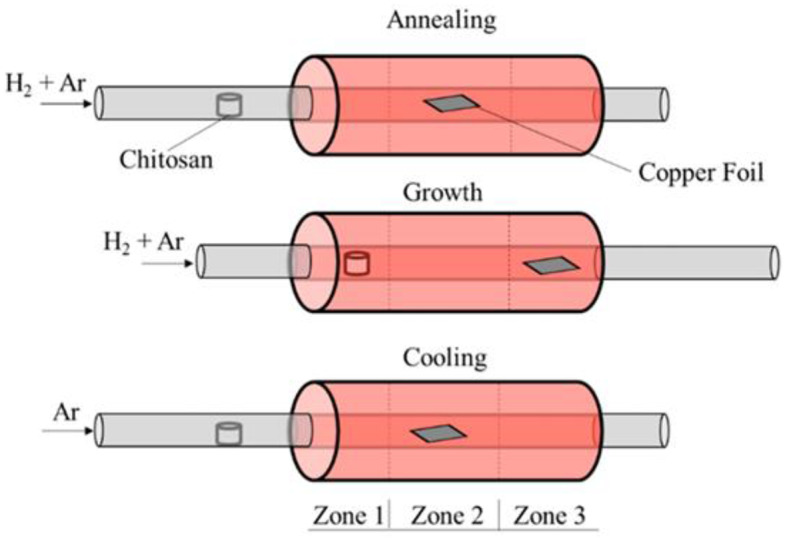
Schematic representation of the N-GQD fabrication *via* chemical vapor deposition (CVD) process; reproduced from Kumar et al. (2018) with permission from the American Chemical Society.

Chemical vapor deposition (CVD) is a well-known approach to prepare 2D graphene. In a CVD technique, the flow rate of the hydrogen (H_2_) and carbon source, growth time, temperature, and the surface morphology of the substrate, are the key parameters, which determine the size of the ultimate product. By tuning these parameters, the nucleation rate of the graphene could be speeded up to exceed the growth rate, leading to a decrease in the size of the final graphene product. The CVD-grown GQDs were first prepared by Fan et al. using copper foil as a substrate and methane as a carbon source, respectively (Fan et al., 2013). In their synthesis experiment, an oxidized surface layer on the copper foil was first removed by cleaning with alcohol and HCl, followed by thermal treatment at 1,000°C in a furnace under a continuous supply of H_2_ and Ar at a flow rate of 10 ml/min and 200 ml/min, respectively. The H_2_ supply was turned off after 40 min, while the H_2_ residues remaining in the reaction tube was removed by supplying Ar for a further 10 min. Afterward, the methane gas (CH_4_) was supplied for only 3 s at a flow rate of 2 ml/min, followed by cooling of the copper foil in an Ar environment. The resultant GQDs showed broad size distribution (5–15 nm) as indicated by the DLS analysis, whereas the height profile (1–3 nm) suggested the formation of few-layer-thick GQDs. Later, GQDs were prepared without any metal catalyst by Ding et al. using hexagonal boron nitride as a substrate (Ding, 2014). By tuning the ratio of different gases, e.g., Ar:CH_4_:H_2_, the GQDs were fabricated under constant reaction time, which possessed a different number of graphene and exhibited thickness-dependent PL in the visible region. Following the CVD approach, the direct formation of single crystalline GQDs on silicon wafer was also demonstrated (Huang et al., 2016). The as-fabricated GQDs were round shaped, 5–10 nm in size, and 2 nm thick, while they possessed an average thickness of 1.2 nm, which suggested single- to few-layer-thick GQDs. Recently, Nessim's group used chitosan, a non-toxic and cheap biopolymer, as a source of C and N to fabricate N-GQDs (Kumar et al., 2018). The DLS analysis (10–15 nm) and the AFM height profile (2–5 nm) suggested the formation of multilayer-thick N-GQDs. They proposed that during the synthesis process, the chitosan first decomposed into N-containing compounds, followed by the adsorption and nucleation of the HCN species to form photoluminescent N-GQDs (Figure 8). Interestingly, the PL emission in the visible region highlighted the potential applications of N-doped GQDs in nano-optoelectronic.

## GQDs for Bioimaging Applications

Previously, organic dyes and inorganic semiconductor QD-based fluorophores have been usually applied for cellular visualization and bioimaging, respectively. However, the photobleaching and the low extinction coefficient of organic dyes, and the poor water solubility and the intrinsic toxicity of the semiconductor QDs, are the main obstacles toward their practical bioimaging applications. Being a 0D member of the carbon family, GQDs hold great promise to actively substitute these fluorophores owing to the tunable and strong PL, photostability, excellent biocompatibility, and effective renal clearance, thus offering unprecedented opportunities for bioimaging (Zheng et al., 2015). In the following sub-sections, the potential bioimaging applications of GQDs, including fluorescence imaging, two-photon imaging, magnetic resonance imaging, and dual-modal imaging, will be discussed.

### Fluorescence Imaging

Since the first demonstration of fluorescent GQDs by Pan et al. (2010), GQDs have been actively developed as a fluorescent probe for monitoring the cellular dynamics as well as *in vitro* and *in vivo* tumor imaging. A redox-sensitive fluorescent probe based on GQDs was devised by Li et al. (2016a). By using this probe, the reductive or oxidative stress-induced dynamic changes in the intracellular redox state were monitored in real time. Notably, the potential of GQDs for determining the cellular dynamics was demonstrated in this study for the first time. Besides the conjugation of GQDs with the recognition elements or proteins, Chen's group later reported the functionalization of GQDs with monosaccharide sugar to determine the trafficking and the overall distribution of cell surface carbohydrate receptor (Chen et al., 2017b). Recently, the same group further performed the real-time estimation of change in the level of intracellular hydrogen sulfide (H_2_S) in live cells (Li et al., 2018b). Specifically, the surface functionalization of the GQDs with dinitrophenyl (an electron-withdrawing group) significantly quenched the PL of GQDs due to the light-induced electron transfer, whereas the cleavage of these groups by H_2_S recovered the GQD PL. Hence, a positive correlation has been noticed between the PL intensity and the intracellular H_2_S level. Compared to the previously developed GQD-based fluorescent probes, the designed fluorescence turn-on strategy ensures the selective determination of H_2_S.

**Figure 9:**
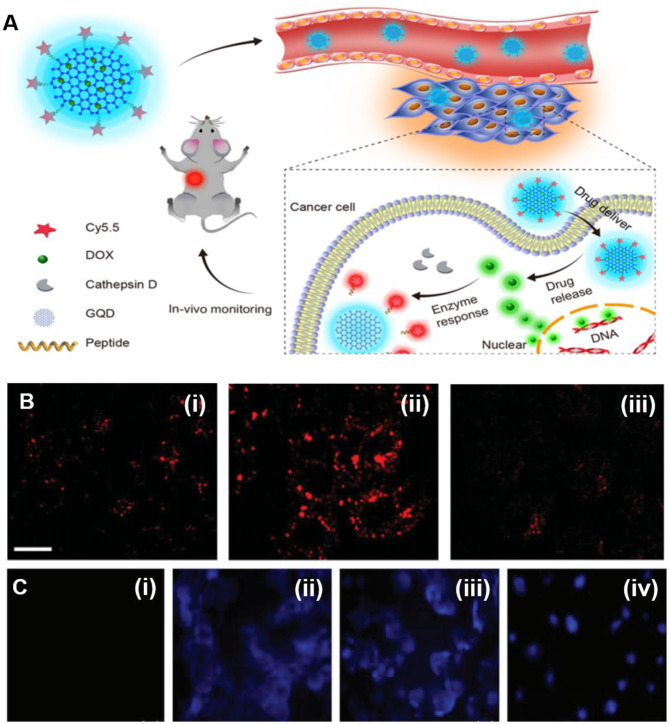
**(A)** Strategy of *in vivo* monitoring of drug and tumor therapy by GQD-based theranostic agent; reproduced from Ding et al. (2017) with permission from the American Chemical Society. **(B)** Confocal fluorescence (FL) images of HeLa cells incubated with GQDs (i), PNF-GQDs (ii), and CO-7 cells incubated with protein nanofiber-conjugated GQDs (PNF-GQDs) (PNF-GQDs) (iii); reproduced from Su et al. (2015) with permission from Wiley. **(C)** Confocal FL images of BMSC (i), MCF7 (ii), MDA-MB-231 (iii), and SKOV3 (iv) under ultraviolet (UV) light irradiation, reproduced from Zhang et al. (2019a) with permission from Wiley.

Instead of monitoring the cellular dynamics, Gao et al. presented polyethyleneimine (PEI)-coated GQDs for *in vitro* tumor cell imaging (Gao et al., 2017). Depending upon the molecular weight (MW) of the PEI, the prepared GQDs exhibited red, yellow, and blue emission, allowing multicolor imaging of U87 tumor cells *in vitro*. They suggested that the coating of PEI not only determined the core structure of GQDs but also altered the energy gap, resulting in multicolor emissive GQDs. Instead of larger and few-layer-thick GQDs, monolayer ultrasmall GQDs were prepared following the pyrolysis approach, showing excitation wavelength-independent blue PL with a QY of 3.6%. Notably, due to the ultrasmall size, GQDs effectively penetrated into the nuclei of HeLa cells as monitored by tracking the blue fluorescence of GQDs during FL imaging (Hong et al., 2018). Considering the water solubility, excellent biocompatibility, and non-toxicity of GQDs, Ding et al. developed a GQD-based theranostic nanoagent loaded with doxorubicin (DOX) (Ding et al., 2017). The internalization of the nanoagent was tracked by the blue fluorescence emitted from the GQDs, while due to the close proximity, the DOX fluorescence was significantly quenched by GQDs. However, a bright green fluorescence is observed from DOX after internalization, which indicated the effective release of DOX from the nanoagent, resulting in substantial chemotherapeutic killing and significant tumor inhibition (Figure 9A).

Meanwhile, the release of red fluorescent Cy 5.5 dye from the nanoagent in response to the overexpression of cathepsin D molecule further confirmed the higher chemotherapeutic killing. Besides the non-selective uptake and cellular imaging, protein nanofiber-conjugated GQDs (PNF-GQDs) offered targeted fluorescence imaging due to the attached RGD receptor as a targeting moiety (Su et al., 2015). Owing to the effective targeting, HeLa cells exhibited far bright fluorescence signal of PNF-GQDs than CO-7 cells as evidenced by confocal microscopy, suggesting the preferential cellular uptake capacity of targeted PNF-GQD probe (Figure 9B). On the other hand, the strong electrostatic interaction between positively charged PNF and negatively charged cellular membrane greatly facilitated the efficient internalization, resulting in a five-fold higher cellular uptake of PNF-GQDs than GQDs alone. Subsequently, folic acid-conjugated GQDs (FA-GQDs) were also reported by Zhang et al. for targeted FL imaging (Zhang et al., 2019a). The time-dependent enhanced fluorescence of FA-GQDs was observed in SKOV3 cells (Figure 9C), which confirmed the selective internalization due to FA targeting, whereas the confocal fluorescence microscopy indicated the positive correlation between the internalization of FA-GQDs and the expression of cell surface FA receptor. Though an enhanced targeting imaging has been achieved, Bansal et al. reported that the conjugation of targeting molecules decreased the PL yield of GQDs (Bansal et al., 2019). They developed plain GQDs conjugated with either biosurfactant or FA receptor. The PL of plain GQDs significantly reduced from 12.8 to 10.4% for biosurfactant-GQDs and 9.08% for FA-GQDs. They suggested that the chemical interactions and local electric field occurred on the GQD surface during bioconjugation leading to a change in the electronic energy of GQDs, resulting in a decreased photoluminescence QY of conjugated GQDs.

**Figure 10:**
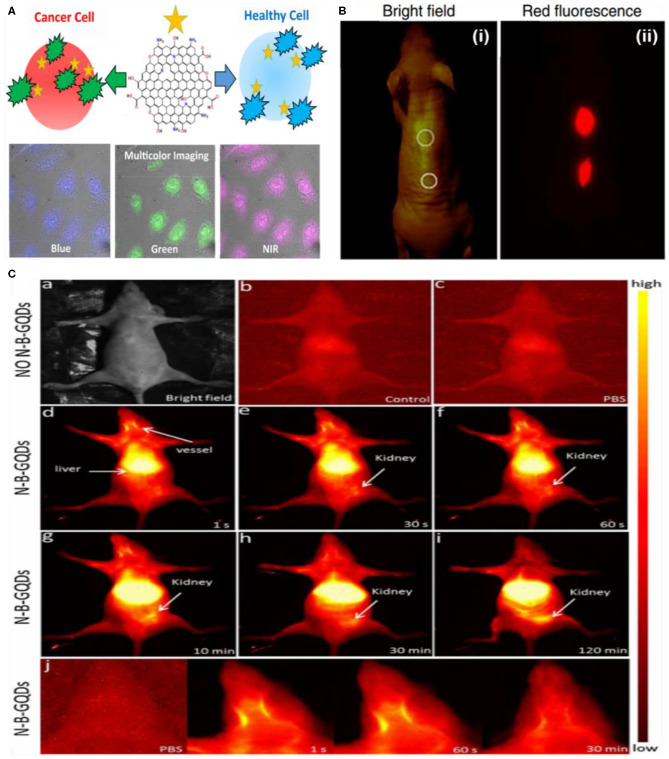
**(A)** Representation of heteroatom-doped GQDs for multicolor imaging and cancer cell detection; reproduced from Campbell et al. (2019) with permission from the American Chemical Society. **(B)** Bright-field (i) and (ii) *in vivo* red-fluorescence image of GQDs after subcutaneous injection; reproduced from Ge et al. (2014) with permission from Nature Publishing Group. **(C)** NIR-II *in vivo* imaging of live mice in supine position; reproduced from Wang et al. (2019b) with permission from Elsevier. (a) Digital image of a nude mouse. (b,c) NIR-II imaging of a control either without injection or with only PBS. (d–i) NIR-II imaging of nude mice at mentioned time points after an *i.v*. injection of N,B co-doped GQDs (1 mg/ml, 200 L). (j) Higher magnified NIR-II images captured at indicated time points, showing blood vessels in the head. The PBS group was used as a control.

In contrast to GQDs, heteroatom-doped GQDs have also been employed for cellular imaging. For instance, Wang's group reported the efficient labeling of HepG2 cells by green fluorescent N-doped GQDs (Li et al., 2018a). In addition, N-GQDs also served as a fluorescent probe for optical sensing of formaldehyde due to the redox-sensitive fluorescence turn on/off. Similarly, B-doped GQDs with excitation-independent PL were prepared by Hai et al. following the one pot acid-free approach (Hai et al., 2015). A bright blue PL under 360-nm excitation was observed in Hela cells incubated with B-GQDs, which suggested the potential of as-obtained B-GQDs for cellular imaging. Later, Wang et al. presented orange fluorescent phosphorus-doped GQDs (P-GQDs) and blue fluorescent B-GQDs, for *in vitro* imaging (Wang et al., 2019a). Owing to the matched energy level, they suggested the rapid energy transfer from B-GQDs to P-GQDs, which led to an increase in the QY of P-GQDs. Recently, visible light-emitted S-GQDs have also been used for cellular imaging (Jin et al., 2018). In contrast, Campbell et al. reported multicolor emissive S-doped, N-doped, and B,N-co-doped GQDs for both visible and NIR-I imaging *in vitro* (Campbell et al., 2019). Under different excitation wavelengths, these doped GQDs emitted blue (450 nm), green (535 nm), and red (750 nm) light (Figure 10A). Notably, this multicolor emission is attributed to the quantum size of GQDs and the electronic state or the arrangements of the surface defects. Moreover, the pH-dependent fluorescence emission of these GQDs also allowed the ratiometric detection of healthy (HEK-293 cell) and tumor cells (HeLa and MCF-7 cell). Similarly, heteroatom co-doped GQDs such as P,N and Fe,N co-doped GQDs were also demonstrated for cellular imaging by different research groups (Ananthanarayanan et al., 2015; Gao et al., 2018). Besides *in vitro* cellular imaging, Wang's group developed red fluorescent GQDs and demonstrated NIR-I *in vivo* imaging (Ge et al., 2014). After sub-cutaneous injection, GQDs exhibited much higher FL intensity at the injection site than mouse skin without any apparent decay (Figure 10B). However, the fabrication process of GQDs is quite lengthy, laborious, and complicated. Compared to NIR-I, *in vivo* imaging in the NIR-II region was recently established by Zhang and coworkers using N,B-co-doped GQDs, which exhibited broad PL emission (950–1,100 nm) (Wang et al., 2019b). Under 808-nm laser excitation, a bright fluorescence signal of intravenously injected N,B-co-doped GQDs was clearly observed in the liver and kidneys of the mice. Moreover, the designed GQDs also allowed an efficient visualization of the blood vessels (Figure 10C). Collectively, N-B-GQDs showed great potential as an NIR-II nanoprobe for organ and vasculature imaging *in vivo*.

### Two-Photon Imaging

Two-photon fluorescence imaging (TPFI) has attained enormous attention because of larger tissue penetration, low signal-to-noise ratio, minimum background autofluorescence, less photobleaching, and reduced photoinduced toxicity, thus holding great promise in biomedical research and diagnostics than single-photon Fl imaging (Yoo et al., 2015). Compared to continuous wave (CW) excited one-photon Fl imaging (OPFI), TPFI offered several advantages: (1) the detailed monitoring of deeply occurring biological activities within the body; (2) high spatiotemporal resolution and reduced photobleaching due to the femtosecond pulsed laser excited two photons *via* non-linear excitation; (3) Fl imaging of deeply residing organelles/tissues as well as the diagnosis of deep-seated tumors owing to the two-photon excitation in both first and second biological window (700–1,350 nm) (Lin et al., 2016).

**Figure 11:**
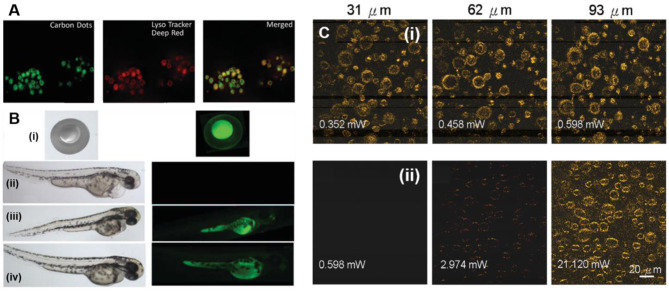
**(A)** *In vitro* co-localization study using Lyso Tracker to monitor the internalization of the GQDs in RAW cells. **(B)** Bright-field (left) and FL images (right) of zebra fish embryos and larvae treated with GQD solution for 30 min, and then imaging at different time-points (i) 1 hpf, (ii,iii) 72 hpf, and (iv) 96 hpf. (ii) the control; reproduced from Singh et al. (2019) with permission from the Royal Society of Chemistry. **(C)** Depth and laser power-dependent TPFL images of N-GQDs labeled (i) and unlabeled A431 cells (ii); reproduced from Wu et al., 2018) with permission from the Royal Society of Chemistry.

Recently, GQDs have emerged as a promising candidate for TPFI and surpassed the conventional two-photon fluorophores because of high two-photon absorption (TPA) cross-section and excellent photostability. For instance, Pu's group reported the ultrasonic preparation of TP fluorescent GQDs with a TPA cross-section of 47,903 Goppert–Mayer units (GM, and 1 GM = 10^−50^ cm^4^ s photon^−1^) in the NIR-I region and a QY of 0.187 (Kuo et al., 2016). Under TP excitation (TPE, 800 nm) at a power of 0.74 mW, the TP luminescence emitted from GQDs revealed the clear localization of GQD-treated gram-positive and gram-negative bacteria, even at a depth of 75 μm. Along with TPFI, the designed GQDs also induced substantial photodynamic antibacterial killing under pulsed laser excitation (2.64 mW) for only 15 s, proving their potential as a photosensitizer for TP photodynamic antibacterial therapy (TP-PDT). Similarly, amino-functionalized N-GQDs (amino-N-GQDs), as a dual-modal agent for TPFI and TP-activated antibacterial PDT of multi-drug resistant bacteria, were developed by Kuo et al. (2018). Importantly, amino functionalization and the N-doping changed the intrinsic electronic and optical properties of GQDs, resulting in high TPA cross-section (54,356 GM) and a QY of 0.33, of the as-obtained amino-N-GQDs. In a subsequent study, amino and sulfo co-functionalized GQDs were also demonstrated for TPFI (Wang et al., 2018b). Though these GQDs showed little low TPA cross-section (38,000 GM), the ultra-high stability due to the edge functionalization suggested their suitability for long-term TP cellular imaging. Recently, Singh et al. reported on *in vitro* and *in vivo* TPFI using hydrothermally synthesized GQDs from neem extract (Singh et al., 2019). A bright green luminescence of GQDs from RAW cells under TPE at 900 nm indicated the internalization of GQDs in lysosome, as further evidenced by LysoTracker (Figure 11A). In addition, *in vivo* two-photon microscopy (TPM) of zebrafish embryo and larvae showed strong fluorescence of GQDs in a yolk sac region compared with other tissues, confirming the potential of GQDs as a contrast agent for TPFI of the digestive system (Figure 11B). Peptide-conjugated GQDs also showed enhanced penetration into MCF-7 cells as monitored by the TPFI, but they showed 6,500 GM TPA cross-section, which is much lower than other GQD-based TPFI contrast agents (Sapkota et al., 2017). In addition to TP cellular imaging, GQDs have also been employed by different researchers for the *in vitro* and *in vivo* TPFI of biomolecules. For example, Zhao et al. demonstrated the *in vitro* and *in vivo* TPFI of H_2_O_2_ (Zhao et al., 2016b), while Tan's group used GQDs for the *in vitro* TPFI of ascorbic acid (Feng et al., 2017).

Like GQDs, heteroatom-doped GQDs also showed potential as a contrast probe for TPFI. For example, Liu et al. prepared N-GQDs *via* solvothermal approach for deep TPFI of cells/tissues (Liu et al., 2013b). In this work, they used DMF as both an N source and a solvent, and the resultant N-GQDs showed high TPA cross-section (48,000 GM). Under femtosecond pulsed laser (800 nm) excitation, HeLa cells stained with GQDs exhibited bright blue fluorescence. Interestingly, compared to the OPFI (400 μm), the TP fluorescence signal was detected even at a tissue thickness of 1,800 μm, which suggested the potential of N-GQDs for deep cellular imaging at least within 800–1,500 μm thickness. Later, single-layer-thick (0.83 nm) polymer-conjugated N-GQDs were developed by Wu et al. for TPFI (Wu et al., 2018). As N-doping and the surface passivation of GQDs by polymer conjugation significantly trapped the emissive energy on the surface, the N-GQDs exhibited high TPA cross-section (57,980.1 GM) and impressive QY (0.573), along with high PL emission intensity in an acidic condition. Owing to high TPA cross-section and TP excitation (TPE) at 800 nm, N-GQDs allowed TPFI in the near-infrared I (NIR-I) region as 35-fold brighter TP signal was observed in N-GQD-labeled A431 skin cells than unlabeled cells under a low TPE power (0.598 mW) (Figure 11C).

### Magnetic Resonance Imaging (MRI)

Being a highly sensitive and non-invasive technique, MRI has emerged as a state-of-the-art imaging modality, offering high spatiotemporal resolution and deep tissue penetration (Vijayalaxmi et al., 2015). Impressively, these features allowed quantitative interrogation of diverse cellular events, tissues dynamics, and cellular anatomy. On the other hand, compared to ionizing radiation-based imaging modalities such as positron emission tomography (PET) and computed tomography (CT), MRI is a non-ionizing radiation-based approach, thereby avoiding the radiation-induced damages and toxicity. Besides the potential advantages, the long operational time of MRI for signal acquisition as well as its low sensitivity (~10^−3^-10^−5^ mol/L) required careful considerations for practical clinical applications (Revia and Zhang, 2016).

**Figure 12:**
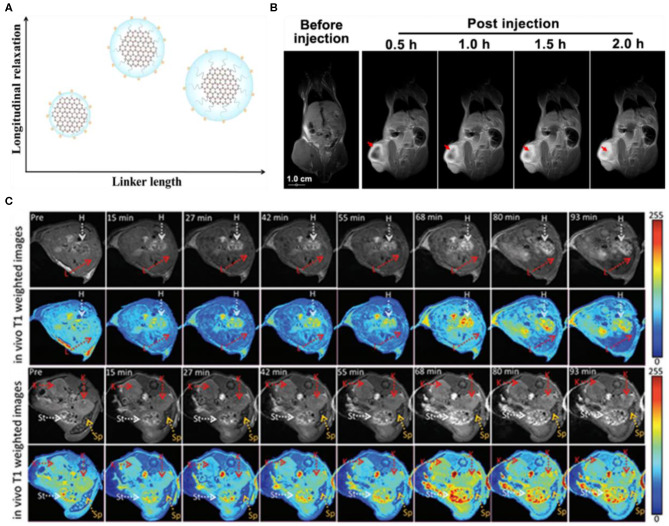
**(A)** Influence of polyethylene glycol (PEG) chains on the longitudinal relaxivity. **(B)** *In Vivo T*_1_-weighted MR images of A549 tumor-bearing mice before and after injection of hyaluronic acid-targeted PGQD (PGQD-HA); reproduced from Yang et al. (2019) with permission from the American Chemical Society. **(C)** *In Vivo T*_1_-weighted MR images of mice before and after *i.v*. injection of B-GQDs; reproduced from Wang et al. (2017) with permission from Wiley. The arrows represented different organs: heart (H), kidney (K), stomach (St), and spleen (Sp).

In MRI, the *T*_1_ relaxation time (longitudinal) and the *T*_2_ relaxation time (transverse) of the magnetic moment of water proton are usually determined. It has been observed that the magnetic moment of water proton is environment dependent, and thus, different attempts have been made to enhance the relaxivity, which directly led to improve either *T*_1_ or *T*_2_ contrast. Generally, gadolinium (Gd^3+^) and iron oxide were employed as a *T*_1_ and *T*_2_ contrast agents, providing bright and dark MRI images, respectively. However, these contrast agents are more or less toxic due to the non-specific conjugation with biological molecules. In addition, the *T*_1_ contrast agent (Gd^3+^) exhibited little selectivity, low relaxivity, and short circulation time, which significantly decreased the efficiency of MRI. Mostly, Gd^3+^ was incorporated into different carriers to enhance the relaxivity and delayed the circulation time (Wang and Zhou, 2016). Among different carriers, GQD is an attractive nanocarrier owing to its biocompatibility, water solubility, and non-toxicity. Recently, Yang et al. developed paramagnetic GQDs (PGQDs) by incorporating polyethylene glycol (PEG) functionalized Gd-1,4,7,10-tetraazacyclododecane-1,4,7,10-tetraacetic acid (Gd-DOTA) complex (Yang et al., 2019). Different PEG chains were used for functionalization, and the highest longitudinal relaxivity was achieved from GQD-PEG_12_-Gd, which was about 16-fold higher than the commercial MRI contrast agent (Gd-DTPA). They suggested that the PEG chains strongly influenced the Gd^3+^ rotation, and thus, the longitudinal relaxivity could be effectively regulated by controlling the chain length (Figure 12A). After intravenous (*i.v*.) injection into A549 tumor-bearing mice, the hyaluronic acid-targeted PGQDs (PGQDs-HA) selectively entered into the tumor and showed significantly enhanced MRI signal after 2 h post-injection (Figure 12B). In addition, owing to the DOX loading, PGQDs induced substantial chemotherapeutic killing, which suggested that MRI-guided cancer therapy could be achieved using PGQD-based theranostic platform. Though an improved MRI efficiency has been demonstrated, the toxicity associated with Gd raises serious safety concerns.

Alternatively, Zhang's group reported paramagnetic metal-free B-GQDs as an MRI contrast agent with a longitudinal relaxivity (*r*_1_) of 18.277 mm^−1^ s^−1^ (Wang et al., 2017). The paramagnetic characteristic was attributed to the introduction of vacancies and elemental boron *via* doping. Meanwhile, the much higher *r*_1_ compared with the un-doped GQDs (0.0038 mm^−1^ s^−1^) and even commercial Gd-DTPA (5.39 mm^−1^ s^−1^) confirmed the doping enhanced longitudinal relaxivity. Moreover, the B-GQDs also showed substantial time-dependent contrast enhancement *in vivo* after subcutaneous injection (Figure 12C). Thus, B-GQDs are a standalone and safer alternative to previously developed Gd-based contrast agents, holding great potential for clinical MRI.

### Dual-Modal Imaging

The concept of integrating multiple imaging modalities into one system has gained much popularity, recently (Fan et al., 2017). By combining the advantages of individual imaging modality, dual-modal imaging offers highly efficient and accurate tumor diagnosis (Huang et al., 2014). On the other hand, compared to the administration of different contrast agents for individual imaging, the introduction of multiple imaging functionalities into a single agent greatly avoids the stress on the body as well as promotes an effective clearance of such dual-modal contrast agent (Cheng et al., 2012).

**Figure 13:**
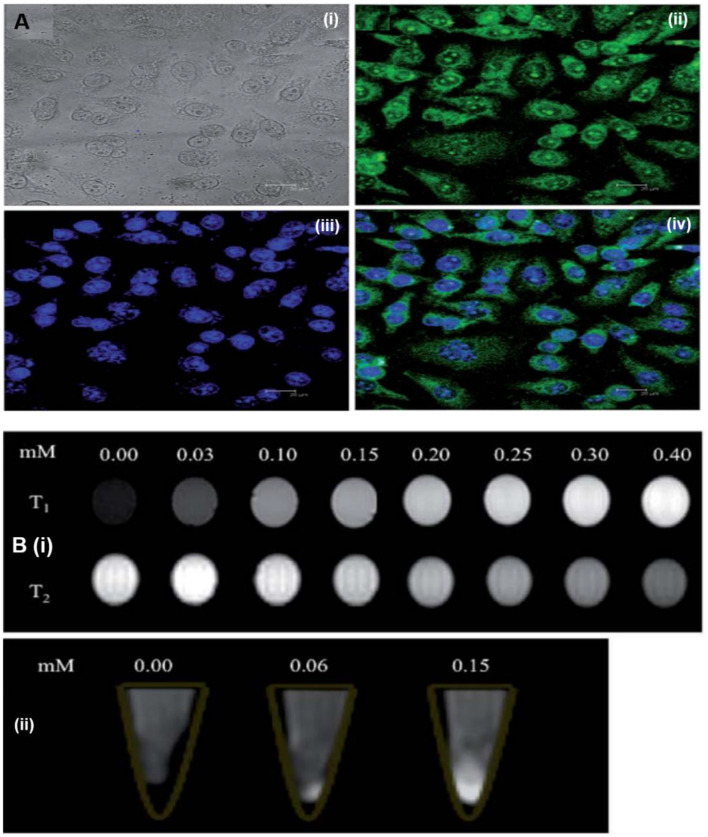
**(A)** *In Vitro* internalization of folate-GdGQDs in HeLa cells. **(B)** *T*_1_and *T*_2_-weighted MRI images of diethylenetriaminepentaacetic acid gadolinium and then decorated with FA receptor (folate-GdGQDs) at various Gd concentrations (i) and *T*_1_*-*weighted MRI images of HeLa cells incubated with various concentration of folate-GdGQDs (ii); reproduced from Huang et al. (2015) with permission from the Royal Society of Chemistry.

Owing to the intrinsic fluorescence, GQDs have been actively integrated with other imaging modalities such as MRI, photoacoustic imaging (PAI), and optical coherence tomography (OCT), and thus, the different GQD-based contrast agents have been developed for dual-modal Fl/MRI, Fl/PAI, and Fl/OCT imaging. For example, Huang et al. developed paramagnetic GQD-based theranostic platform for dual-modal (Fl/MRI) imaging and targeted chemotherapy (Huang et al., 2015). In their work, the paramagnetic GQDs were first fabricated by the covalent conjugation of diethylenetriaminepentaacetic acid gadolinium (Gd-GQDs) and then decorated with FA receptor (folate-GdGQDs) to achieve targeted imaging guided chemotherapy by the loaded DOX. Under visible light, confocal microscopic (CLSM) analysis revealed the bright green fluorescence of folate-GdGQDs internalized into HeLa cells, confirming their selective uptake *via* receptor-mediated endocytosis as well as *in vitro* imaging capacity (Figure 13A). Meanwhile, the higher longitudinal relaxivity (*r*_1_, 11.49 mm^−1^ s^−1^) and transverse relaxivity (*r*_2_, 28.79 mm^−1^ s^−1^) owing to Gd conjugation allowed the enhanced *T*_1_ and *T*_2_-weighted MRI (Figure 13B). Along with dual-modal imaging, folate-GdGQDs selectively delivered the DOX, leading to significant chemotherapeutic killing *in vitro*. Hence, folate-GdGQDs showed great promise as a nanotheranostic agent for dual-modal imaging-guided targeted cancer chemotherapy.

Subsequently, instead of G-doped GQDs, superparamagnetic GQDs (MGQDs) were developed by Justin et al. for dual-modal Fl/MRI (Justin et al., 2016). Iron oxide nanoparticles (Fe_3_O_4_) were incorporated onto GQDs *via* a facile approach, and the resultant MGQDs exhibited PL emission at 398 nm and a *T*_2_ relaxation time of 4.16 mm^−1^ s^−1^ with a strong dependence on Fe concentration. Meanwhile, a concentration-dependent enhanced PL signal of MGQDs was observed from glioblastoma cells, which suggested an efficient *in vitro* imaging. Besides Fl/MRI contrast agent, MGQDs also served as a drug nanocarrier as well as a photothermal agent, presenting dual-modal imaging-guided synergistic chemo/photothermal tumor therapy. In another study, targeted dual-modal Fl/MRI-guided chemotherapy by Fe_3_O_4_@SiO_2_@GQD-FA was also demonstrated (Su et al., 2017). The PL emission at 460 nm and an enhanced transverse relaxation time (*r*_2_, 62.8 mm^−1^ s^−1^) for *T*_2_-weighted MRI enabled an efficient guidance of chemotherapy by dual-modal Fl/MRI *in vitro*.

**Figure 14:**
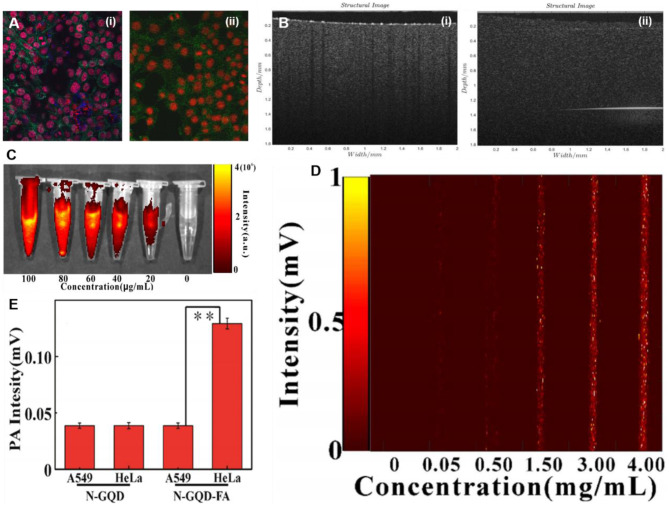
**(A)** Visible light, confocal microscopic (CLSM) images of superparamagnetic GQDs (MGQDs) labeled (i) and unlabeled (ii) 3T3 cells. **(B)** magnetomotive optical coherence tomography (OCT) images of MGQDs labeled (i) and unlabeled (ii) 3T3 cells; reproduced from Li et al. (2019) with permission from Wiley. **(C,D)** Concentration-dependent enhanced near-infrared (NIR) fluorescence as well as photoacoustic imaging (PAI) signal. **(E)** *In Vitro* PAI intensity of FA-N-GQDs and N-GQDs in both HeLa cells and A549 cells, respectively; reproduced from Xuan et al. (2018) with permission from IOP Publishing. **means *P* value is 0.05.

In contrast to Fl/MRI, Li et al. reported on superparamagnetic GQDs (MGQDs) as a dual-modal contrast agent for Fl/optical coherence tomography imaging (Fl/OCT) (Li et al., 2019). The MGQDs internalized into 3T3 exhibited blue fluorescence as revealed by *in vitro* CLSM imaging (Figure 14A), while for OCT, the MGQD-labeled cells were placed on an agar gel to mimic the human soft tissues, and then imaged by an in-house magnetomotive OCT system. Compared to unlabeled 3T3 cells, a significant magnetomotive signal (320 Hz) was observed from MGQD-labeled cells, which allowed the successful cellular visualization by OCT (Figure 14B). Hence, the intrinsic fluorescence and superparamagnetic property make MGQDs a promising contrast agent for dual-modal Fl/OCT imaging. Recently, using ^131^I-labeled FA-GQDs, Wei's group demonstrated targeted single-photon emission computed tomography (SPECT) imaging *in vivo* (Wang et al., 2019c). Owing to FA targeting, the ^131^I-labeled FA-GQDs were preferentially uptaken by the HeLA cells, while after *i.v*. into mice, the *in vivo* SPECT imaging revealed their higher accumulation in the liver and tumor region, compared to those of non-conjugated GQDs. Importantly, the biodistribution study confirmed their effective distribution and rapid metabolization after 48-h post-injection, suggesting the rapid clearance from the body to ensure the non-toxicity of the GQDs. Owing to efficient SPECT imaging, the designed GQDs would be a suitable candidate for the SPECT imaging of FA-overexpressed tumor. Dual-modal Fl/PAI imaging using N-GQDs was reported by Xuan et al. for the first time (Xuan et al., 2018). The developed N-GQDs exhibited fluorescence emission in both visible and NIR regions. Meanwhile, a concentration-dependent enhanced NIR fluorescence as well as PAI signal were observed (Figures 14C,D), which suggested the linear relationship (*R*^2^ = 0.9969). After FA functionalization, FA-N-GQDs were preferably uptaken by the HeLa cells than A549 cells as revealed by *in vitro* PAI (Figure 14E) and triggered significant photothermal destruction of tumor cells due to the PTT effect. These findings strongly conferred that the targeted tumor PTT could be achieved under the guidance of dual-modal Fl/PAI by FA-conjugated N-GQDs.

## Toxicity of GQDs

The inherent toxicity of inorganic nanomaterials presents prominent obstacles to their potential biomedical applications. Therefore, the *in vitro* and *in vivo* toxicity of GQDs should be clearly understood to advance their practical bioimaging applications. Though GQDs belong to a family of carbon nanomaterial, which predicts their low cytotoxicity (Kakran et al., 2011), the detailed toxicity investigations prior to their introduction into the biological systems are mandatory to ensure their non-toxic biological effects. It has been noticed that the surface charge, functional groups, and the size of GQDs determine the toxicity of the QDs (Schroeder et al., 2016). Compared to pristine GQDs, PEGylated GQDs showed less toxicity due to the biocompatible PEG molecule. Notably, the PEG surface functionalization led to an increase in the size of the resultant GQDs as well as it imparts a hydrophilic character, resulting in reduced toxic effects and safer biological imaging applications (Chandra et al., 2014). We have presented the *in vitro* and *in vivo* toxicity of GQDs in Table 1. For thorough understanding, the readers are referred to the comprehensive and state-of-the-art recently published reviews (Schroeder et al., 2016; Wang et al., 2016a; Selestin Raja et al., 2019), which provided the elaborative and in-detail review of GQD toxicity.

**Table 1:** Toxicity of graphene quantum dots (GQDs) and doped-GQDs.

**Material**	**Cells**	**Assay**	**Toxicity**	**Incubation time**	**References**
GQDs (2 mg/ml)	MCF-7, Hela, MCF-10A	MTT	>95%	24 h	Roy et al., 2015
GQDs (160 μg/ml) (640 μg/ml)	Hela, A549	MTT LDH	>95% >85%	24 h	Chong et al., 2014
GQDs (100 μg/ml)	Hela	CKK-8	90%	24 h	Jiang et al., 2015
GQDs (200 μg/ml)	A549	MTT	>80%	24 h	Yuan et al., 2014
GQDs (500 μg/ml)	KB, MDA-MB231, A549 MDCK	MTT LDH	>95%	21 days/24 h	Nurunnabi et al., 2013
GQDs (100 μg/ml)	A549	MTT	80%	24 h	Sun et al., 2013
GQDs	MGC-803 MCF-7	MTT	GQDs < GO	3 days	Wu et al., 2013
GQDs (200 μg/ml)	Stem cells	MTT	>70%	24 h	Zhang et al., 2012
GQDs (100 μg/ml)	Stem cells	MTT	61%	24 h	Qiu et al., 2016
GQDs (200 μg/ml)	THP-1 macrophages	MTT	82.5%	24 h	Qin et al., 2015b
GQDs (400 μg/ml)	MG-63 MC3T3	MTT	>80%	24 h	Zhu et al., 2011
GQDs (200 μg/ml)	RSC96	MTT	70%	24 h	Zhu et al., 2015c
B-GQDs (4 mg/ml)	Hela	MTT	87%	24 h	Hai et al., 2015
N-GQDs	Red blood cells (RBC)	Hemolysis ATP	N-GQDs < GO	12 h	Wang et al., 2015

Chong et al. determined the toxicity of PEG-GQDs onto HeLa cells and A549 cells by LDH assay and WST-1, respectively (Chong et al., 2014). They suggested no apparent cytotoxicity as more than 80% of the cell viability was recorded for both HeLa and A549 cells even at a high concentration (160 μg/ml for HeLa and 640 μg/ml for A549). Moreover, compared to the control, a comparable ROS level and LDH were observed, which confirmed that PEG-GQDs neither disrupt the cell membrane integrity nor induce oxidative stress. In contrast to cellular toxicity, Wu et al. conducted the genotoxicity investigations of GQDs by performing cell cycle analysis (Wu et al., 2013). They found that GQDs slightly damaged the cellular DNA of MCF-7 and MGC 803 cells, but this was still far superior than GO, which triggered significant cell accumulation in the sub-G1 phase, resulting in increased cell apoptosis.

Apart from *in vitro* testing, Nurunnabi et al. demonstrated that *i.v*.-injected carboxylated GQDs exert very low toxicity and organ damage even after 21 days as verified by complete blood counts, histological analysis, and blood biochemistry (Nurunnabi et al., 2013). In another report, Chong et al. suggested that intravenous and intraperitoneal injection of multiple doses of PEG-GQDs (20 mg/kg every second day) into mice for 14 days did not induce any noticeable toxicity compared to the control (Chong et al., 2014). Similarly, no apparent toxicity of GQDs and gadolinium-doped FA functionalized GQDs (Gd-FA-GQDs) were observed in zebrafish (Huang et al., 2015). However, Gd-FA-GQDs induced little toxicity at a higher concentration (500 μg/ml), which is attributed to the release of Gd ions from the GQDs. This implies that the toxicity of the doping element as well as the stability of the overall GQD complex should be taken into consideration to ensure biological safety.

## Current Challenges and Future Prospects

Despite the significant advancements and the proven remarkable advantages, the potential bioimaging applications of GQDs have not been fully explored yet owing to certain unresolved challenges. (1) Although GQDs have been fabricated by a number of methods as discussed in the Synthesis Strategies of GQDs section, the controlled formation of high-quality single-layer GQDs with narrow size distribution is still a challenging task. Meanwhile, most of these reported methods usually suffer from long reaction time, toxic organic solvents, etc., and provide considerably low product yield. As the optical and electronic properties of GQDs strongly depend on their size and shape, the controlled formation of GQDs is an ideal way to enhance their intrinsic physicochemical properties; therefore, the exploration of a controllable, high-yield, and environment friendly method for GQD fabrication is highly desirable yet quite advantageous for the large-scale industrial production of high-quality GQDs for biomedical applications. (2) The designed GQDs exhibited very low QY for green and red fluorescence, which is even lower than the organic fluorophores and conventional semiconductor QDs. This low QY especially in the NIR region presented a potential barrier toward the practical applications of GQDs for tumor imaging. Meanwhile, the alteration of optical properties by surface passivation or heteroatom doping could enhance the fluorescence QY of GQDs. On the other hand, the poor understanding regarding the PL mechanism of GQDs seriously limited the further improvement in QY. Despite the several proposed mechanisms of PL, including surface state, quantum confinement effect, surface modification, edge state, doping, size effect, etc., a clear and detailed mechanism of PL with sufficient interpretation is still missing. Hence, the integration of experimental investigations with theoretical measurements is greatly needed to provide a deeper and thorough understanding of the PL mechanism. (3) Most of the reported GQDs exhibited narrow spectral PL emission either in the blue or green fluorescence, thus seriously suffering from low penetration depth and high tissue scattering, respectively. However, the formation of GQDs having an NIR I/II emission is a formidable challenge as the QY of GQDs in the NIR region is considerably low, which limited the further advancement of GQDs as a contrast agent for bioimaging. Owing to the minimum auto-fluorescence and low light absorption by the tissues in the NIR region, GQDs with NIR-I or NIR-II emission will be a potential candidate for *in vivo* imaging of deep-seated tumors, whereas with the rapid development of new and innovative fabrication strategies, the NIR I/II-emitted GQDs with high QY could be expected in the near future for precise and accurate tumor diagnosis.

## Conclusion

In this review, we have attempted to provide a comprehensive account of the latest cutting-edge advancements in GQD research with a special emphasis on their bioimaging applications. The recent progress in fabrication strategies including top–down and bottom–up has been critically reviewed, following an in-detail discussion on the potential *in vitro* and *in vivo* bioimaging applications of GQDs. A comparative and balanced viewpoint has been given, which may assist to overcome the existing odds, and facilitates the design of next-generation GQD-based contrast agents for clinical diagnostics. We believe that this timely rigorous account offers an in-depth understanding, which will promote more exciting and innovative developments in the future, leading to shift in the GQDs from bench to bedside.

## Author Contributions

MY wrote the manuscript. GH helped in designing the schematic. MY, JL, and PH discussed and revised the manuscript. All authors contributed to manuscript revision, read, and approved the submitted version.

## Conflict of Interest

The authors declare that the research was conducted in the absence of any commercial or financial relationships that could be construed as a potential conflict of interest.

## References

[B1] Ahirwar S. Mallick S. Bahadur D. (2017). Electrochemical method to prepare graphene quantum dots and graphene oxide quantum dots. ACS Omega 2, 8343–8353. 10.1021/acsomega.7b01539 31457373 PMC6645081

[B2] Ananthanarayanan A. Wang X. Routh P. Sana B. Lim S. Kim D.-H. (2014). Facile synthesis of graphene quantum dots from 3D graphene and their application for Fe^3+^ sensing. Adv. Funct. Mater. 24, 3021–3026. 10.1002/adfm.201303441

[B3] Ananthanarayanan A. Wang Y. Routh P. Sk M. A. Than A. Lin M.. (2015). Nitrogen and phosphorus co-doped graphene quantum dots: synthesis from adenosine triphosphate, optical properties, and cellular imaging. Nanoscale 7, 8159–8165. 10.1039/C5NR01519G 25875153

[B4] Baker M. (2010). Nanotechnology imaging probes: smaller and more stable. Nat. Methods 7, 957–962. 10.1038/nmeth1210-957

[B5] Bansal S. Singh J. Kumari U. Kaur I. P. Barnwal R. Kumar R.. (2019). Development of biosurfactant-based graphene quantum dot conjugate as a novel and fluorescent theranostic tool for cancer. Int. J. Nanomed. 14, 809–818. 10.2147/IJN.S188552 30774335 PMC6354693

[B6] Benítez-Martínez S. Valcárcel M. (2015). Graphene quantum dots in analytical science, TrAC Tr. Anal. Chem. 72, 93–113. 10.1016/j.trac.2015.03.020

[B7] Boonta W. Talodthaisong C. Sattayaporn S. Chaicham C. Chaicham A. Sahasithiwat S. (2020). The synthesis of nitrogen and sulfur co-doped graphene quantum dots for fluorescence detection of cobalt(ii) ions in water, mater. Chem. Front. 4, 507–516. 10.1039/C9QM00587K

[B8] Campbell E. Hasan M. T. Gonzalez Rodriguez R. Akkaraju G. R. Naumov A. V. (2019). Doped graphene quantum dots for intracellular multicolor imaging and cancer detection. ACS Biomater. Sci. Eng. 5, 4671–4682. 10.1021/acsbiomaterials.9b00603 33448839

[B9] Chandra A. Deshpande S. Shinde D. B. Pillai V. K. Singh N. (2014). Mitigating the cytotoxicity of graphene quantum dots and enhancing their applications in bioimaging and drug delivery. ACS Macro Lett. 3, 1064–1068. 10.1021/mz500479k 35610793

[B10] Chen F. Gao W. Qiu X. Zhang H. Liu L. Liao P. (2017a). Graphene quantum dots in biomedical applications: recent advances and future challenges. Front. Lab. Med. 1, 192–199. 10.1016/j.flm.2017.12.006

[B11] Chen G. Zhuo Z. Ni K. Kim N. Y. Zhao Y. Chen Z.. (2015). Rupturing C60 molecules into graphene-oxide-like quantum dots: structure, photoluminescence, and catalytic application. Small 11, 5296–5304. 10.1002/smll.201501611 26287442

[B12] Chen J. Than A. Li N. Ananthanarayanan A. Zheng X. Xi F. (2017b). Sweet graphene quantum dots for imaging carbohydrate receptors in live cells. FlatChem 5, 25–32. 10.1016/j.flatc.2017.08.006

[B13] Chen W. Li D. Tian L. Xiang W. Wang T. Hu W. (2018). Synthesis of graphene quantum dots from natural polymer starch for cell imaging. Green Chem. 20, 4438–4442. 10.1039/C8GC02106F

[B14] Cheng Z. Al Zaki A. Hui J. Z. Muzykantov V. R. Tsourkas A. (2012). Multifunctional nanoparticles: cost versus benefit of adding targeting and imaging capabilities. Science 338, 903–910. 10.1126/science.1226338 23161990 PMC3660151

[B15] Chong Y. Ma Y. Shen H. Tu X. Zhou X. Xu J.. (2014). The *in vitro* and *in vivo* toxicity of graphene quantum dots. Biomaterials 35, 5041–5048. 10.1016/j.biomaterials.2014.03.021 24685264

[B16] Cirone J. Ahmed S. R. Wood P. C. Chen A. (2019). Green synthesis and electrochemical study of cobalt/graphene quantum dots for efficient water splitting. J. Phys. Chem. C 123, 9183–9191. 10.1021/acs.jpcc.9b00951

[B17] Ding H. Zhang F. Zhao C. Lv Y. Ma G. Wei W.. (2017). Beyond a carrier: graphene quantum dots as a probe for programmatically monitoring anti-cancer drug delivery, release, and response. ACS Appl. Mater. Interf. 9, 27396–27401. 10.1021/acsami.7b08824 28782357

[B18] Ding X. (2014). Direct synthesis of graphene quantum dots on hexagonal boron nitride substrate. J. Mater. Chem. C 2, 3717–3722. 10.1039/C4TC00298A

[B19] Dong Y. Pang H. Ren S. Chen C. Chi Y. Yu T. (2013). Etching single-wall carbon nanotubes into green and yellow single-layer graphene quantum dots. Carbon 64, 245–251. 10.1016/j.carbon.2013.07.059

[B20] Fan L. Zhu M. Lee X. Zhang R. Wang K. Wei J. (2013). Direct synthesis of graphene quantum dots by chemical vapor deposition. Part. Syst. Charact. 30, 764–769. 10.1002/ppsc.201300125

[B21] Fan W. Yung B. Huang P. Chen X. (2017). Nanotechnology for multimodal synergistic cancer therapy. Chem. Rev. 117, 13566–13638. 10.1021/acs.chemrev.7b00258 29048884

[B22] Fan Z. Li S. Yuan F. Fan L. (2015). Fluorescent graphene quantum dots for biosensing and bioimaging. RSC Adv. 5, 19773–19789. 10.1039/C4RA17131D

[B23] Fan Z. Li Y. Li X. Fan L. Zhou S. Fang D. (2014). Surrounding media sensitive photoluminescence of boron-doped graphene quantum dots for highly fluorescent dyed crystals, chemical sensing and bioimaging. Carbon 70, 149–156. 10.1016/j.carbon.2013.12.085

[B24] Farain Md Noor N. Saiful Badri M. A. Salleh M. M. Umar A. A. (2018). Synthesis of white fluorescent pyrrolic nitrogen-doped graphene quantum dots. Opt. Mater. 83, 306–314. 10.1016/j.optmat.2018.06.040

[B25] Feng L.-L. Wu Y.-X. Zhang D.-L. Hu X.-X. Zhang J. Wang P.. (2017). Near infrared graphene quantum dots-based two-photon nanoprobe for direct bioimaging of endogenous ascorbic acid in living cells. Anal. Chem. 89, 4077–4084. 10.1021/acs.analchem.6b04943 28281746

[B26] Gao T. Wang X. Yang L.-Y. He H. Ba X.-X. Zhao J.. (2017). Red, yellow, and blue luminescence by graphene quantum dots: syntheses, mechanism, and cellular imaging. ACS Appl. Mater. Interf. 9, 24846–24856. 10.1021/acsami.7b05569 28675929

[B27] Gao X. X. Zhou X. Ma Y. F. Wang C. P. Chu F. X. (2018). A fluorometric and colorimetric dual-mode sensor based on nitrogen and iron co-doped graphene quantum dots for detection of ferric ions in biological fluids and cellular imaging. New J. Chem. 42, 14751–14756. 10.1039/C8NJ01805G

[B28] Ge J. Lan M. Zhou B. Liu W. Guo L. Wang H.. (2014). A graphene quantum dotphotodynamic therapy agent with high singlet oxygen generation. Nat. Commun. 5:4596. 10.1038/ncomms5596 25105845 PMC4143951

[B29] Habiba K. Bracho-Rincon D. P. Gonzalez-Feliciano J. A. Villalobos-Santos J. C. Makarov V. I. Ortiz D.. (2015). Synergistic antibacterial activity of PEGylated silver-graphene quantum dots nanocomposites. Appl. Mater. Today 1, 80–87. 10.1016/j.apmt.2015.10.00126766909

[B30] Hai X. Mao Q.-X. Wang W.-J. Wang X.-F. Chen X.-W. Wang J.-H. (2015). An acid-free microwave approach to prepare highly luminescent boron-doped graphene quantum dots for cell imaging. J. Mater. Chem. B 3, 9109–9114. 10.1039/C5TB01954K 32263124

[B31] Halder A. Godoy-Gallardo M. Ashley J. Feng X. Zhou T. Hosta-Rigau L. (2018). One-pot green synthesis of biocompatible graphene quantum dots and their cell uptake studies. ACS Appl. BioMater. 1, 452–461. 10.1021/acsabm.8b00170 35016368

[B32] Haque E. Kim J. Malgras V. Reddy K. R. Ward A. C. You J. (2018). Recent advances in graphene quantum dots: synthesis, properties, and applications. Small Methods 2:1800050 10.1002/smtd.201800050

[B33] Hassan M. Haque E. Reddy K. R. Minett A. I. Chen J. Gomes V. G. (2014). Edge-enriched graphene quantum dots for enhanced photo-luminescence and supercapacitance. Nanoscale 6, 11988–11994. 10.1039/C4NR02365J 25178096

[B34] Hong G.-L. Zhao H.-L. Deng H.-H. Yang H.-J. Peng H. Liu Y.-H.. (2018). Fabrication of ultra-small monolayer graphene quantum dots by pyrolysis of trisodium citrate for fluorescent cell imaging. Inter. J. Nanomed. 13, 4807–4815. 10.2147/IJN.S168570 30197516 PMC6113908

[B35] Huang C.-L. Huang C.-C. Mai F.-D. Yen C.-L. Tzing S.-H. Hsieh H.-T.. (2015). Application of paramagnetic graphene quantum dots as a platform for simultaneous dual-modality bioimaging and tumor-targeted drug delivery. J. Mater. Chem. B 3, 651–664. 10.1039/C4TB01650E 32262348

[B36] Huang H. Yang S. Li Q. Yang Y. Wang G. You X.. (2018). Electrochemical cutting in weak aqueous electrolytes: the strategy for efficient and controllable preparation of graphene quantum dots. Langmuir 34, 250–258. 10.1021/acs.langmuir.7b03425 29249142

[B37] Huang K. Lu W. Yu X. Jin C. Yang D. (2016). Highly pure and luminescent graphene quantum dots on silicon directly grown by chemical vapor deposition. Part. Syst. Charact. 33, 8–14. 10.1002/ppsc.201500132

[B38] Huang P. Rong P. Jin A. Yan X. Zhang M. G. Lin J.. (2014). Dye-loaded ferritin nanocages for multimodal imaging and photothermal therapy. Adv. Mater. 26, 6401–6408. 10.1002/adma.201400914 25123089 PMC4215197

[B39] Jiang D. Chen Y. Li N. Li W. Wang Z. Zhu J.. (2015). Synthesis of luminescent graphene quantum dots with high quantum yield and their toxicity study. PLOS ONE 10:e0144906. 10.1371/journal.pone.0144906 26709828 PMC4699207

[B40] Jin K. Gao H. Lai L. Pang Y. Zheng S. Niu Y. (2018). Preparation of highly fluorescent sulfur doped graphene quantum dots for live cell imaging. J. Luminescence 197, 147–152. 10.1016/j.jlumin.2018.01.028

[B41] Justin R. Tao K. Román S. Chen D. Xu Y. Geng X. (2016). Photoluminescent and superparamagnetic reduced graphene oxide-iron oxide quantum dots for dual-modality imaging, drug delivery and photothermal therapy. Carbon 97, 54–70. 10.1016/j.carbon.2015.06.070

[B42] Kakran M. Sahoo N. Bao H. Pan Y. Li L. (2011). Functionalized graphene oxide as nanocarrier for loading and delivery of ellagic acid. Curr. Med. Chem. 18, 4503–4512. 10.2174/092986711797287548 21864287

[B43] Kumar S. Aziz S. K. T. Girshevitz O. Nessim G. D. (2018). One-step synthesis of N-doped graphene quantum dots from chitosan as a sole precursor using chemical vapor deposition. J. Phys. Chem. C 122, 2343–2349. 10.1021/acs.jpcc.7b05494

[B44] Kundu S. Ghosh S. Fralaide M. Narayanan T. N. Pillai V. K. Talapatra S. (2015a). Fractional photo-current dependence of graphene quantum dots prepared from carbon nanotubes. Phys. Chem. Chem. Phys. 17, 24566–24569. 10.1039/C5CP03306C 26351706

[B45] Kundu S. Yadav R. M. Narayanan T. N. Shelke M. V. Vajtai R. Ajayan P. M.. (2015b). Synthesis of N, F and S co-doped graphene quantum dots. Nanoscale 7, 11515–11519. 10.1039/C5NR02427G 26087457

[B46] Kuo W.-S. Chang C.-Y. Chen H.-H. Hsu C.-L. L. Wang J.-Y. Kao H.-F.. (2016). Two-photon photoexcited photodynamic therapy and contrast agent with antimicrobial graphene quantum dots. ACS Appl. Mater. Interf. 8, 30467–30474. 10.1021/acsami.6b12014 27753472

[B47] Kuo W.-S. Shao Y.-T. Huang K.-S. Chou T.-M. Yang C.-H. (2018). Antimicrobial amino-functionalized nitrogen-doped graphene quantum dots for eliminating multidrug-resistant species in dual-modality photodynamic therapy and bioimaging under two-photon excitation. ACS Appl. Mater. Interf. 10, 14438–14446. 10.1021/acsami.8b01429 29620851

[B48] Kwon W. Kim Y.-H. Lee C.-L. Lee M. Choi H. C. Lee T.-W.. (2014). Electroluminescence from graphene quantum dots prepared by amidative cutting of tattered graphite. Nano Lett. 14, 1306–1311. 10.1021/nl404281h 24490804

[B49] Lee N. E. Lee S. Y. Lim H. S. Yoo S. H. Cho S. O. (2020). A novel route to high-quality graphene quantum dots by hydrogen-assisted pyrolysis of silicon carbide Nanomaterials 10:277. 10.3390/nano10020277 32041275 PMC7075118

[B50] Lee S. H. Kim D. Y. Lee J. Lee S. B. Han H. Kim Y. Y.. (2019). Synthesis of single-crystalline hexagonal graphene quantum dots from solution chemistry. Nano Lett. 19, 5437–5442. 10.1021/acs.nanolett.9b01940 31274324

[B51] Li H. He X. Liu Y. Huang H. Lian S. Lee S.-T. (2011). One-step ultrasonic synthesis of water-soluble carbon nanoparticles with excellent photoluminescent properties. Carbon 49, 605–609. 10.1016/j.carbon.2010.10.004

[B52] Li H.-J. Sun X. Xue F. Ou N. Sun B.-W. Qian D.-J. (2018a). Redox induced fluorescence on-off switching based on nitrogen enriched graphene quantum dots for formaldehyde detection and bioimaging. ACS Sustain. Chem. Eng. 6, 1708–1716. 10.1021/acssuschemeng.7b02941

[B53] Li K. Liu W. Ni Y. Li D. Lin D. Su Z.. (2017). Technical synthesis and biomedical applications of graphene quantum dots. J. Mater. Chem. B 5, 4811–4826. 10.1039/C7TB01073G 32263997

[B54] Li L. Wu G. Yang G. Peng J. Zhao J. Zhu J.-J. (2013a). Focusing on luminescent graphene quantum dots: current status and future perspectives Nanoscale 5, 4015–4039. 10.1039/c3nr33849e 23579482

[B55] Li L.-L. Ji J. Fei R. Wang C.-Z. Lu Q. Zhang J.-R. (2012a). A facile microwave avenue to electrochemiluminescent two-color graphene quantum dots. Adv. Funct. Mater. 22, 2971–2979. 10.1002/adfm.201200166

[B56] Li N. Than A. Chen J. Xi F. Liu J. Chen P. (2018b). Graphene quantum dots based fluorescence turn-on nanoprobe for highly sensitive and selective imaging of hydrogen sulfide in living cells. Biomater. Sci. 6, 779–784. 10.1039/C7BM00818J 29134987

[B57] Li N. Than A. Sun C. Tian J. Chen J. Pu K.. (2016a). Monitoring dynamic cellular redox homeostasis using fluorescence-switchable graphene quantum dots. ACS Nano 10, 11475–11482. 10.1021/acsnano.6b07237 28024361

[B58] Li Q. Zhang S. Dai L. Li L.-S. (2012b). Nitrogen-doped colloidal graphene quantum dots and their size-dependent electrocatalytic activity for the oxygen reduction reaction. J. Am. Chem. Soc. 134, 18932–18935. 10.1021/ja309270h 23126520

[B59] Li R. Liu Y. Li Z. Shen J. Yang Y. Cui X.. (2016b). Bottom-up fabrication of single-layered nitrogen-doped graphene quantum dots through intermolecular carbonization arrayed in a 2D plane. Chem. A Eur. J. 22, 272–278. 10.1002/chem.201503191 26593633

[B60] Li W. Li M. Liu Y. Pan D. Li Z. Wang L. (2018c). Three minute ultrarapid microwave-assisted synthesis of bright fluorescent graphene quantum dots for live cell staining and white LEDs. ACS Appl. Nano Mater. 1, 1623–1630. 10.1021/acsanm.8b00114

[B61] Li W. Song W. Chen B. Matcher S. J. (2019). Superparamagnetic graphene quantum dots as a dual-modality contrast agent for confocal fluorescence microscopy and magnetomotive optical coherence tomography. J. Biophotonics 12:201800219. 10.1002/jbio.201800219 30191684

[B62] Li X. Lau S. P. Tang L. Ji R. Yang P. (2013b). Multicolour light emission from chlorine-doped graphene quantum dots. J. Mater. Chem. C 1, 7308–7313. 10.1039/c3tc31473a

[B63] Li Y. Liu H. Liu X.-Q. Li S. Wang L. Ma N.. (2016c). Free-radical-assisted rapid synthesis of graphene quantum dots and their oxidizability studies. Langmuir 32, 8641–8649. 10.1021/acs.langmuir.6b02422 27506575

[B64] Li Y. Zhao Y. Cheng H. Hu Y. Shi G. Dai L.. (2012c). Nitrogen-doped graphene quantum dots with oxygen-rich functional groups. J. Am. Chem. Soc. 134, 15–18. 10.1021/ja206030c 22136359

[B65] Lin J. Chen X. Huang P. (2016). Graphene-based nanomaterials for bioimaging. Adv. Drug Deliv. Rev. 105, 242–254. 10.1016/j.addr.2016.05.013 27233213 PMC5039069

[B66] Liu F. Jang M.-H. Ha H. D. Kim J.-H. Cho Y.-H. Seo T. S. (2013a). Facile synthetic method for pristine graphene quantum dots and graphene oxide quantum dots: origin of blue and green luminescence. Adv. Mater. 25, 3657–3662. 10.1002/adma.201300233 23712762

[B67] Liu Q. Guo B. Rao Z. Zhang B. Gong J. R. (2013b). Strong two-photon-induced fluorescence from photostable, biocompatible nitrogen-doped graphene quantum dots for cellular and deep-tissue imaging. Nano Lett. 13, 2436–2441. 10.1021/nl400368v 23675758

[B68] Liu R. Wu D. Feng X. Müllen K. (2011). Bottom-up fabrication of photoluminescent graphene quantum dots with uniform morphology. J. Am. Chem. Soc. 133, 15221–15223. 10.1021/ja204953k 21894989

[B69] Lu J. Tang M. Zhang T. (2019). Review of toxicological effect of quantum dots on the liver. J. Appl. Toxicol. 39, 72–86. 10.1002/jat.3660 30091143

[B70] Lu J. Yeo P. S. E. Gan C. K. Wu P. Loh K. P. (2011). Transforming C60 molecules into graphene quantum dots. Nat. Nanotechnol. 6, 247–252. 10.1038/nnano.2011.30 21423185

[B71] Luo Z. Qi G. Chen K. Zou M. Yuwen L. Zhang X. (2016). Microwave-assisted preparation of white fluorescent graphene quantum dots as a novel phosphor for enhanced white-light-emitting diodes. Adv. Funct. Mater. 26, 2739–2744. 10.1002/adfm.201505044

[B72] Maiti S. Kundu S. Roy C. N. Das T. K. Saha A. (2017). Synthesis of excitation independent highly luminescent graphene quantum dots through perchloric acid oxidation. Langmuir 33, 14634–14642. 10.1021/acs.langmuir.7b02611 29172551

[B73] Michalet X. Pinaud F. F. Bentolila L. A. Tsay J. M. Doose S. Li J. J.. (2005). Quantum dots for live cells, *in vivo* imaging, and diagnostics. Science 307, 538–544. 10.1126/science.1104274 15681376 PMC1201471

[B74] Nair R. V. Thomas R. T. Sankar V. Muhammad H. Dong M. Pillai S. (2017). Rapid, acid-free synthesis of high-quality graphene quantum dots for aggregation induced sensing of metal ions and bioimaging. ACS Omega 2, 8051–8061. 10.1021/acsomega.7b01262 30023571 PMC6045375

[B75] Nurunnabi M. Khatun Z. Huh K. M. Park S. Y. Lee D. Y. Cho K. J.. (2013). *In vivo* biodistribution and toxicology of carboxylated graphene quantum dots. ACS Nano 7, 6858–6867. 10.1021/nn402043c 23829293

[B76] Ozhukil Valappil M. K. Pillai V. Alwarappan S. (2017). Spotlighting graphene quantum dots and beyond: synthesis, properties and sensing applications. Appl. Mater. Today 9, 350–371. 10.1016/j.apmt.2017.09.002

[B77] Pan D. Zhang J. Li Z. Wu M. (2010). Hydrothermal route for cutting graphene sheets into blue-luminescent graphene quantum dots. Adv. Mater. 22, 734–738. 10.1002/adma.200902825 20217780

[B78] Peng J. Gao W. Gupta B. K. Liu Z. Romero-Aburto R. Ge L.. (2012). Graphene quantum dots derived from carbon fibers. Nano Lett. 12, 844–849. 10.1021/nl2038979 22216895

[B79] Qi B.-P. Zhang X. Shang B.-B. Xiang D. Zhang S. (2018). Solvothermal tuning of photoluminescent graphene quantum dots: from preparation to photoluminescence mechanism. J. Nanoparticle Res. 20:20 10.1007/s11051-018-4123-8

[B80] Qin Y. Cheng Y. Jiang L. Jin X. Li M. Luo X. (2015a). Top-down strategy toward versatile graphene quantum dots for organic/inorganic hybrid solar cells. ACS Sus. Chem. Eng. 3, 637–644. 10.1021/sc500761n

[B81] Qin Y. Zhou Z.-W. Pan S.-T. He Z.-X. Zhang X. Qiu J.-X.. (2015b). Graphene quantum dots induce apoptosis, autophagy, and inflammatory response via p38 mitogen-activated protein kinase and nuclear factor-κB mediated signaling pathways in activated THP-1 macrophages. Toxicology 327, 62–76. 10.1016/j.tox.2014.10.011 25446327

[B82] Qiu J. Li D. Mou X. Li J. Guo W. Wang S.. (2016). Effects of graphene quantum dots on the self-renewal and differentiation of mesenchymal stem cells. Adv. Healt. Mater. 5, 702–710. 10.1002/adhm.201500770 26833812

[B83] Qu D. Sun Z. Zheng M. Li J. Zhang Y. Zhang G. (2015). Three colors emission from S,N co-doped graphene quantum dots for visible light H2 production and bioimaging. Adv. Opt. Mater. 3, 360–367. 10.1002/adom.201400549

[B84] Ren Q. Ga L. Ai J. (2019). Rapid synthesis of highly fluorescent nitrogen-doped graphene quantum dots for effective detection of ferric ions and as fluorescent ink. ACS Omega 4, 15842–15848. 10.1021/acsomega.9b01612 31592454 PMC6776961

[B85] Revia R. A. Zhang M. (2016). Magnetite nanoparticles for cancer diagnosis, treatment, and treatment monitoring: recent advances. Mater. Today 19, 157–168. 10.1016/j.mattod.2015.08.022 27524934 PMC4981486

[B86] Roy P. Periasamy A. P. Lin C.-Y. Her G.-M. Chiu W.-J. Li C.-L.. (2015). Photoluminescent graphene quantum dots for *in vivo* imaging of apoptotic cells. Nanoscale 7, 2504–2510. 10.1039/C4NR07005D 25569453

[B87] Sapkota B. Benabbas A. Lin H.-Y. G. Liang W. Champion P. Wanunu M. (2017). Peptide-decorated tunable-fluorescence graphene quantum dots. ACS Appl. Mater. Interf. 9, 9378–9387. 10.1021/acsami.6b16364 28252932

[B88] Schroeder K. L. Goreham R. V. Nann T. (2016). Graphene quantum dots for theranostics and bioimaging. Pharmaceu. Res. 33, 2337–2357. 10.1007/s11095-016-1937-x 27207272

[B89] Selestin Raja I. P. Song S.-J. Kang M. Lee Y. Kim B. Hong S. W.. (2019). Toxicity of zero- and one-dimensional carbon nanomaterials. Nanomaterials 9:1214. 10.3390/nano9091214 31466309 PMC6780407

[B90] Shen H. Zhang L. Liu M. Zhang Z. (2012). Biomedical applications of graphene. Theranostics 2, 283–294. 10.7150/thno.3642 22448195 PMC3311234

[B91] Shen J. Zhu Y. Chen C. Yang X. Li C. (2011). Facile preparation and upconversion luminescence of graphene quantum dots. Chem. Commun. 47, 2580–2582. 10.1039/C0CC04812G 21173992

[B92] Shin Y. Park J. Hyun D. Yang J. Lee J.-H. Kim J.-H.. (2015). Acid-free and oxone oxidant-assisted solvothermal synthesis of graphene quantum dots using various natural carbon materials as resources. Nanoscale 7, 5633–5637. 10.1039/C5NR00814J 25757839

[B93] Singh H. Sreedharan S. Tiwari K. Green N. H. Smythe C. Pramanik S. K.. (2019). Two photon excitable graphene quantum dots for structured illumination microscopy and imaging applications: lysosome specificity and tissue-dependent imaging. Chem. Commun. 55, 521–524. 10.1039/C8CC08610A 30556083

[B94] Song S. H. Jang M.-H. Chung J. Jin S. H. Kim B. H. Hur S.-H. (2014). Highly efficient light-emitting diode of graphene quantum dots fabricated from graphite intercalation compounds. Adv. Opt. Mater. 2, 1016–1023. 10.1002/adom.201400184

[B95] Su X. Chan C. Shi J. Tsang M.-K. Pan Y. Cheng C.. (2017). A graphene quantum Dot@Fe_3_O_4_@SiO_2_ based nanoprobe for drug delivery sensing and dual-modal fluorescence and MRI imaging in cancer cells. Biosens. Bioelect. 92, 489–495. 10.1016/j.bios.2016.10.076 27839733

[B96] Su Z. Shen H. Wang H. Wang J. Li J. Nienhaus G. U. (2015). Motif-designed peptide nanofibers decorated with graphene quantum dots for simultaneous targeting and imaging of tumor cells. Adv. Funct. Mater. 25, 5472–5478. 10.1002/adfm.201502506

[B97] Sun H. Ji H. Ju E. Guan Y. Ren J. Qu X. (2015). Synthesis of fluorinated and nonfluorinated graphene quantum dots through a new top-down strategy for long-time cellular imaging. Chem. A Eur. J. 21, 3791–3797. 10.1002/chem.201406345 25614445

[B98] Sun Y. Wang S. Li C. Luo P. Tao L. Wei Y.. (2013). Large scale preparation of graphene quantum dots from graphite with tunable fluorescence properties. Phys. Chem. Chem. Phys. 15, 9907–9913. 10.1039/c3cp50691f 23673490

[B99] Tan X. Li Y. Li X. Zhou S. Fan L. Yang S. (2015). Electrochemical synthesis of small-sized red fluorescent graphene quantum dots as a bioimaging platform. Chem. Commun. 51, 2544–2546. 10.1039/C4CC09332A 25567527

[B100] Tang L. Ji R. Li X. Bai G. Liu C. P. Hao J.. (2014). Deep ultraviolet to near-infrared emission and photoresponse in layered N-doped graphene quantum dots. ACS Nano 8, 6312–6320. 10.1021/nn501796r 24848545

[B101] Tian P. Tang L. Teng K. S. Lau S. P. (2018). Graphene quantum dots from chemistry to applications. Mater. Today Chem. 10, 221–258. 10.1016/j.mtchem.2018.09.00727584033

[B102] Tian R. Zhong S. Wu J. Jiang W. Shen Y. Jiang W. (2016). Solvothermal method to prepare graphene quantum dots by hydrogen peroxide. Opt. Mater. 60, 204–208. 10.1016/j.optmat.2016.07.032

[B103] Vijayalaxmia Fatahi M. Speck O. (2015). Magnetic resonance imaging (MRI): a review of genetic damage investigations. Mutat. Res. Rev. Mutat. Res. 764, 51–63. 10.1016/j.mrrev.2015.02.002 26041266

[B104] Wang G. Guo Q. Chen D. Liu Z. Zheng X. Xu A.. (2018a). Facile and highly effective synthesis of controllable lattice sulfur-doped graphene quantum dots via hydrothermal treatment of durian. ACS Appl. Mater. Interf. 10, 5750–5759. 10.1021/acsami.7b16002 29350521

[B105] Wang G. He P. Xu A. Guo Q. Li J. Wang Z.. (2019a). Promising fast energy transfer system between graphene quantum dots and the application in fluorescent bioimaging. Langmuir 35, 760–766. 10.1021/acs.langmuir.8b03739 30485105

[B106] Wang H. Mu Q. Wang K. Revia R. A. Yen C. Gu X.. (2019b). Nitrogen and boron dual-doped graphene quantum dots for near-infrared second window imaging and photothermal therapy. Appl. Mater. Today 14, 108–117. 10.1016/j.apmt.2018.11.011 31538108 PMC6752708

[B107] Wang H. Revia R. Wang K. Kant R. J. Mu Q. Gai Z.. (2017). Paramagnetic properties of metal-free boron-doped graphene quantum dots and their application for safe magnetic resonance imaging. Adv. Mater. 29:1605416. 10.1002/adma.201605416 28026064 PMC5391173

[B108] Wang H. Zhou S. (2016). Magnetic and fluorescent carbon-based nanohybrids for multi-modal imaging and magnetic field/NIR light responsive drug carriers. Biomater. Sci. 4, 1062–1073. 10.1039/C6BM00262E 27184106

[B109] Wang L. Li W. Li M. Su Q. Li Z. Pan D.. (2018b). Ultrastable amine, sulfo cofunctionalized graphene quantum dots with high two-photon fluorescence for cellular imaging. ACS Sus. Chem. Eng. 6, 4711–4716. 10.1021/acssuschemeng.7b0379732262352

[B110] Wang S. Cole I. S. Li Q. (2016a). The toxicity of graphene quantum dots. RSC Adv. 6, 89867–89878. 10.1039/C6RA16516H

[B111] Wang T. Zhu S. Jiang X. (2015). Toxicity mechanism of graphene oxide and nitrogen-doped graphene quantum dots in RBCs revealed by surface-enhanced infrared absorption spectroscopy. Toxicol. Res. 4, 885–894. 10.1039/C4TX00138A

[B112] Wang X. Sun G. Li N. Chen P. (2016b). Quantum dots derived from two-dimensional materials and their applications for catalysis and energy. Chem. Soc. Rev. 45, 2239–2262. 10.1039/C5CS00811E 26848039

[B113] Wang Y. Song H. Wang G. Yang X. Wang J. Wei H. (2019c). 131I-Labeled PEG and folic acid co-functionalized graphene quantum dots for tumor-targeted imaging. J. Radioanal. Nuclear Chem. 319:1119–1125. 10.1007/s10967-019-06434-8

[B114] Wu C. Wang C. Han T. Zhou X. Guo S. Zhang J. (2013). Insight into the cellular internalization and cytotoxicity of graphene quantum dots. Adv. Health. Mater. 2, 1613–1619. 10.1002/adhm.201300066 23703800

[B115] Wu P.-C. Wang J.-Y. Wang W.-L. Chang C.-Y. Huang C.-H. Yang K.-L.. (2018). Efficient two-photon luminescence for cellular imaging using biocompatible nitrogen-doped graphene quantum dots conjugated with polymers. Nanoscale 10, 109–117. 10.1039/C7NR06836K 29211084

[B116] Xu H. Zhou S. Xiao L. Wang H. Li S. Yuan Q. (2015). Fabrication of a nitrogen-doped graphene quantum dots from MOF-derived porous carbon and its application for highly selective fluorescence detection of Fe^3+.^ *J*. Mater. Chem. C 3, 291–297. 10.1039/C4TC01991A

[B117] Xuan Y. Zhang R.-Y. Zhang X.-S. An J. Cheng K. Li C.. (2018). Targeting N-doped graphene quantum dots with high photothermal conversion efficiency for dual-mode imaging and therapy *in vitro*. Nanotechnology 29:355101. 10.1088/1361-6528/aacad0 29873637

[B118] Yan X. Cui X. Li L.-S. (2010). Synthesis of large, stable colloidal graphene quantum dots with tunable size. J. Am. Chem. Soc. 132, 5944–5945. 10.1021/ja1009376 20377260

[B119] Yan Y. Gong J. Chen J. Zeng Z. Huang W. Pu K.. (2019). Recent advances on graphene quantum dots: from chemistry and physics to applications. Adv. Mater. 31:1808283. 10.1002/adma.201808283 30828898

[B120] Yang Y. Chen S. Li H. Yuan Y. Zhang Z. Xie J.. (2019). Engineered paramagnetic graphene quantum dots with enhanced relaxivity for tumor imaging. Nano Lett. 19, 441–448. 10.1021/acs.nanolett.8b04252 30560672

[B121] Ye R. Xiang C. Lin J. Peng Z. Huang K. Yan Z.. (2013). Coal as an abundant source of graphene quantum dots. Nat. Commun. 4:2943. 10.1038/ncomms3943 24309588

[B122] Yin Y. Liu Q. Jiang D. Du X. Qian J. Mao H. (2016). Atmospheric pressure synthesis of nitrogen doped graphene quantum dots for fabrication of BiOBr nanohybrids with enhanced visible-light photoactivity and photostability. Carbon 96, 1157–1165. 10.1016/j.carbon.2015.10.068

[B123] Yoo J. M. Kang J. H. Hong B. H. (2015). Graphene-based nanomaterials for versatile imaging studies. Chem. Soc. Rev. 44, 4835–4852. 10.1039/C5CS00072F 25777530

[B124] Yousaf M. Huang H. Li P. Wang C. Yang Y. (2017). Fluorine functionalized graphene quantum dots as inhibitor against hIAPP amyloid aggregation. ACS Chem. Neurosci. 8, 1368–1377. 10.1021/acschemneuro.7b00015 28230965

[B125] Yu J. Liu S. Chen S. Wang T. (2017). Simultaneous preparation of mesoporous/macroporous graphene aerogels and bright green photoluminescent graphene quantum dots by a simple solvothermal method. Ind. Eng. Chem. Res. 56, 10028–10035. 10.1021/acs.iecr.7b01954

[B126] Yuan X. Liu Z. Guo Z. Ji Y. Jin M. Wang X. (2014). Cellular distribution and cytotoxicity of graphene quantum dots with different functional groups. Nanoscale Res. Lett. 9:108. 10.1186/1556-276X-9-108 24597852 PMC3973856

[B127] Zhang M. Bai L. Shang W. Xie W. Ma H. Fu Y. (2012). Facile synthesis of water-soluble, highly fluorescent graphene quantum dots as a robust biological label for stem cells. J. Mater. Chem. 22, 7461–7467. 10.1039/c2jm16835a

[B128] Zhang Q. Deng S. Liu J. Zhong X. He J. Chen X. (2019a). Cancer-targeting graphene quantum dots: fluorescence quantum yields, stability, and cell selectivity. Adv. Funct. Mater. 29:1805860 10.1002/adfm.201805860

[B129] Zhang R. Ding Z. (2018). Recent advances in graphene quantum dots as bioimaging probes. J. Anal. Testing 2, 45–60. 10.1007/s41664-018-0047-7

[B130] Zhang Y. Li K. Ren S. Dang Y. Liu G. Zhang R. (2019b). Coal-derived graphene quantum dots produced by ultrasonic physical tailoring and their capacity for Cu(II) detection. ACS Sust. Chem. Eng. 7, 9793–9799. 10.1021/acssuschemeng.8b06792

[B131] Zhao J. Tang L. Xiang J. Ji R. Hu Y. Yuan J. (2015). Fabrication and properties of a high-performance chlorine doped graphene quantum dots based photovoltaic detector. RSC Adv. 5, 29222–29229. 10.1039/C5RA02358K

[B132] Zhao L. Zong W. Zhang H. Liu R. (2018). Kidney toxicity and response of selenium containing protein-glutathione peroxidase (Gpx3) to CdTe QDs on different levels. Toxicol. Sci. 168, 201–208. 10.1093/toxsci/kfy297 30535317

[B133] Zhao P. Yang M. Fan W. Wang X. Tang F. Yang C. (2016a). Facile one-pot conversion of petroleum asphaltene to high quality green fluorescent graphene quantum dots and their application in cell imaging. Part. Syst. Charact. 33, 635–644. 10.1002/ppsc.201600070

[B134] Zhao W. Li Y. Yang S. Chen Y. Zheng J. Liu C.. (2016b). Target-activated modulation of dual-color and two-photon fluorescence of graphene quantum dots for *in vivo* imaging of hydrogen peroxide. Anal. Chem. 88, 4833–4840. 10.1021/acs.analchem.6b00521 27072323

[B135] Zhao Y. Wu X. Sun S. Ma L. Zhang L. Lin H. (2017). A facile and high-efficient approach to yellow emissive graphene quantum dots from graphene oxide. Carbon 124, 342–347. 10.1016/j.carbon.2017.09.011

[B136] Zheng X. T. Ananthanarayanan A. Luo K. Q. Chen P. (2015). Glowing graphene quantum dots and carbon dots: properties, syntheses, and biological applications. Small 11, 1620–1636. 10.1002/smll.201402648 25521301

[B137] Zhou C. He X. Ya D. Zhong J. Deng B. (2017). One step hydrothermal synthesis of nitrogen-doped graphitic quantum dots as a fluorescent sensing strategy for highly sensitive detection of metacycline in mice plasma. Sens. Actua. B Chem. 249, 256–264. 10.1016/j.snb.2017.04.092

[B138] Zhou S. Xu H. Gan W. Yuan Q. (2016). Graphene quantum dots: recent progress in preparation and fluorescence sensing applications. RSC Adv. 6, 110775–110788. 10.1039/C6RA24349E

[B139] Zhu C. Yang S. Wang G. Mo R. He P. Sun J.. (2015a). A new mild, clean and highly efficient method for the preparation of graphene quantum dots without by-products. J. Mater. Chem. B 3, 6871–6876. 10.1039/C5TB01093D 32262536

[B140] Zhu C. Yang S. Wang G. Mo R. He P. Sun J. (2015b). Negative induction effect of graphite N on graphene quantum dots: tunable band gap photoluminescence. J. Mater. Chem. C 3, 8810–8816. 10.1039/C5TC01933H

[B141] Zhu S. Zhang J. Qiao C. Tang S. Li Y. Yuan W.. (2011). Strongly green-photoluminescent graphene quantum dots for bioimaging applications. Chem. Commun. 47, 6858–6860. 10.1039/c1cc11122a 21584323

[B142] Zhu S. Zhou N. Hao Z. Maharjan S. Zhao X. Song Y. (2015c). Photoluminescent graphene quantum dots for *in vitro* and *in vivo* bioimaging using long wavelength emission. RSC Adv. 5, 39399–39403. 10.1039/C5RA02961A

[B143] Zhuang Q. Wang Y. Ni Y. (2016). Solid-phase synthesis of graphene quantum dots from the food additive citric acid under microwave irradiation and their use in live-cell imaging. Luminescence 31, 746–753. 10.1002/bio.3019 26310294

[B144] Zhuo S. Shao M. Lee S.-T. (2012). Upconversion and downconversion fluorescent graphene quantum dots: ultrasonic preparation and photocatalysis. ACS Nano 6, 1059–1064. 10.1021/nn2040395 22221037

